# Year-round at-sea distribution and trophic resources partitioning between two sympatric Sulids in the tropical Atlantic

**DOI:** 10.1371/journal.pone.0253095

**Published:** 2021-06-21

**Authors:** Nathalie Almeida, Jaime A. Ramos, Isabel Rodrigues, Ivo dos Santos, Jorge M. Pereira, Diana M. Matos, Pedro M. Araújo, Pedro Geraldes, Tommy Melo, Vitor H. Paiva

**Affiliations:** 1 Department of Life Sciences, University of Coimbra, MARE–Marine and Environmental Sciences Centre, Calçada Martim de Freitas, Coimbra, Portugal; 2 Biosfera Cabo Verde, São Vicente, Cabo Verde; 3 CIBIO/InBIO, Centro de Investigação em Biodiversidade e Recursos Genéticos, Campus Agrário de Vairão, Universidade do Porto, Vairão, Portugal; 4 SPEA–Sociedade Portuguesa para o Estudo das Aves, Lisboa, Portugal; Hawaii Pacific University, UNITED STATES

## Abstract

In the oligotrophic tropical marine environment resources are usually more patchily distributed and less abundant to top predators. Thus, spatial and trophic competition can emerge, especially between related seabird species belonging to the same ecological guild. Here we studied the foraging ecology of two sympatric species–brown booby (BRBO) *Sula leucogaster* (breeding) and red-footed boobies (RFBO) *Sula sula* (non-breeding)–at Raso islet (Cabo Verde), across different seasons. Sexual segregation was only observed during Jun-Oct, when RFBO were present, with larger females BRBO remaining closer to the colonies, while males and RFBO travelled further and exploited different habitats. Overall, species appeared to prefer areas with specific oceanic features, particularly those related with oceanic currents and responsible for enhancing primary productivity in tropical oceanic areas (e.g. Sea Surface Height and Ocean Mixed Layer Thickness). Female BRBOs showed high foraging-site fidelity during the period of sympatry, while exploiting the same prey species as the other birds. However, during the months of co-existence (Jun.-Oct.), isotopic mixing models suggested that female BRBO would consume a higher proportion of epipelagic fish, whereas female RFBO would consume more squid compared to the other birds, possibly due to habitat-specific prey availability and breeding energy-constraints for BRBO. We conclude that divergent parental roles, environmental conditions, habitat preference and competition could be mechanisms simultaneously underlying sexual segregation for BRBO during a period of co-existence, while inter-specific foraging differences appear to be more affected by habitat preference and different breeding stages. These results support previous statements that BRBO can adapt their foraging ecology to different circumstances of environmental conditions and competition, and that marine physical features play an important role in foraging decisions of boobies.

## Introduction

Competition for food resources occurs naturally between organisms living in communities [1], and between close-related species sharing the same geographic area (sympatry) for breeding and/or foraging [2,3]. Higher levels of competition may occur particularly when resources are scarce [4,5], which is the likely scenario in oligotrophic tropical seas [6–9]. As an evolutionary strategy to avoid high levels of competition, allowing species and populations to thrive and co-exist in the same environment, adaptations between and within species are expected to exist, displacing each other in such a manner that each takes possession of a certain niche, in which it has an advantage over its competitor [2,3].

Intra-specific competition can exist in large seabird colonies, especially during breeding seasons when adult seabirds are constrained by central place foraging [4,10], with breeders of some species segregating sexually. This type of segregation can usually be explained by three factors: (1) Anatomic differences between males and females (sexual dimorphism) [11–14], most frequently related to size of seabird species [12,14], influencing flight speed, foraging range and flapping frequency, as well as diving depth and duration induced by body mass [15]; (2) Divergent parental roles, influencing nest fidelity; and (3) nutritional requirements [13]. Thus, some sulids exhibit Reversed Sexual Dimorphism (RSD), where the bigger females are the main chick provisioners and the smaller males invest more in nest attendance and defence [13–18]. Here, size difference, when present, work as a limiting factor, by excluding the smaller gender from productive areas closer to the colony or competing for better resources. Although many RSD sulid species show sexual segregation, this is not always the case [14,19–21], perhaps due to harsher environmental conditions at their colony surroundings, resulting in fish stock depletion, or when broods are larger and demand a higher foraging effort [19].

Boobies and gannets are suitable species to study intra- and inter-specific competition, because most of the colonies around the world are very numerous, reaching numbers as high as 750 000 individuals in some places [22]. Additionally, these species are often found breeding and/ or co-existing in mixed colonies with conspecifics [8,14,17,23,24]. Although this co-existence could lead to inter-specific competition, this may also be an opportunity for foraging information exchange [25,26] to locate ephemeral prey patches in tropical areas, as happens with other conspecifics [18,27]. This could be a potential foraging strategy for individuals to contour their patchily distributed prey, because studies of penguins in different sized colonies, confirmed foraging overlap between individuals of smaller colonies, while individuals from larger colonies segregated in their foraging areas [28], possibly after causing prey depletion [29]. The proposed [30] ‘Halo hypothesis’, suggested that tropical and other seabirds populations are regulated by food supply, creating prey-depleted “halos” near the colonies which affect breeding success. These same density-dependent effects that shape seabird colonies [30], were observed in Cape gannets (*Morus capensis*) and Masked boobies (*Sula dactylatra*) from larger colonies which exhibited a higher foraging effort, translating into higher levels of competition when compared to individuals from smaller colonies [31,32].

Some sulid species can exhibit high repeatability in foraging behaviour [33,34], especially adult breeders, in terms of spatial and trophic ecology [35,36], which is dependent on oceanic physical structures such as fronts, shelf edges, seamounts and other processes that are related to marine productivity [37–39]. However, most studies on marine tropical realms, have connected the occurrence of tropical seabirds, such as boobies, with the presence of sub-surface predators in a ‘facilitated foraging behaviour’ [6], which could translate into low foraging site fidelity due to prey unpredictability [24,40].

At Raso Islet, Cabo Verde, RSD brown boobies (hereafter termed BRBO) *Sula leucogaster* breed and inhabit the islet year-round. Between June–October of each year, a recently established population of red-footed boobies (hereafter termed RFBO) *Sula sula* also inhabit the islet for moulting [41]. This sets the ideal scenario to investigate spatio-temporal foraging, trophic and dietary segregation within and between closely-related species, during different periods of the year, using GPS tracking and stable isotope analysis. Studies between sympatric boobies have long been performed around the world in tropical waters, with results being influenced by colony and species size [17,23,24], productivity and environmental conditions of surrounding waters [21,24,42], fish stock distributions and reproductive behaviour [21,42,43]. Most of these studies, however, have worked with sympatric breeding boobies, because it is much harder to retrieve tracking devices from non-breeding species.

Overall, we expect to observe (1) inter-sexual behavioural and spatial segregation of bigger-sized female BRBO and RFBO performing shorter trips and foraging near the colony, while smaller males should forage at a greater distance, driven by RSD size-based competition; (2) inter-species behavioural and spatial segregation when co-occurring at Raso Islet, due to differences in size and breeding stage, with RFBO being more pelagic and with lower repeatability in foraging behaviour given the absence of breeding duties; (3) the foraging activity of RFBO to be triggered by gradients in environmental predictors (e.g. gradient in depth), which are known to depict oceanic frontal regimes likely occurring in pelagic areas that they might exploit in the absence of breeding duties, while BRBO should rely on local-scale changes of marine productivity patterns (e.g. Sea Surface Temperature), occurring in the colony surroundings, to frequently return to the colony for chick provisioning; (4) broader isotopic niches and a more diverse diet composition of RFBO when compared to BRBO individuals, which may be a reflection of a more generalist and pelagic diet.

## Methods

### Study site and logger deployment

**Fig 1:**
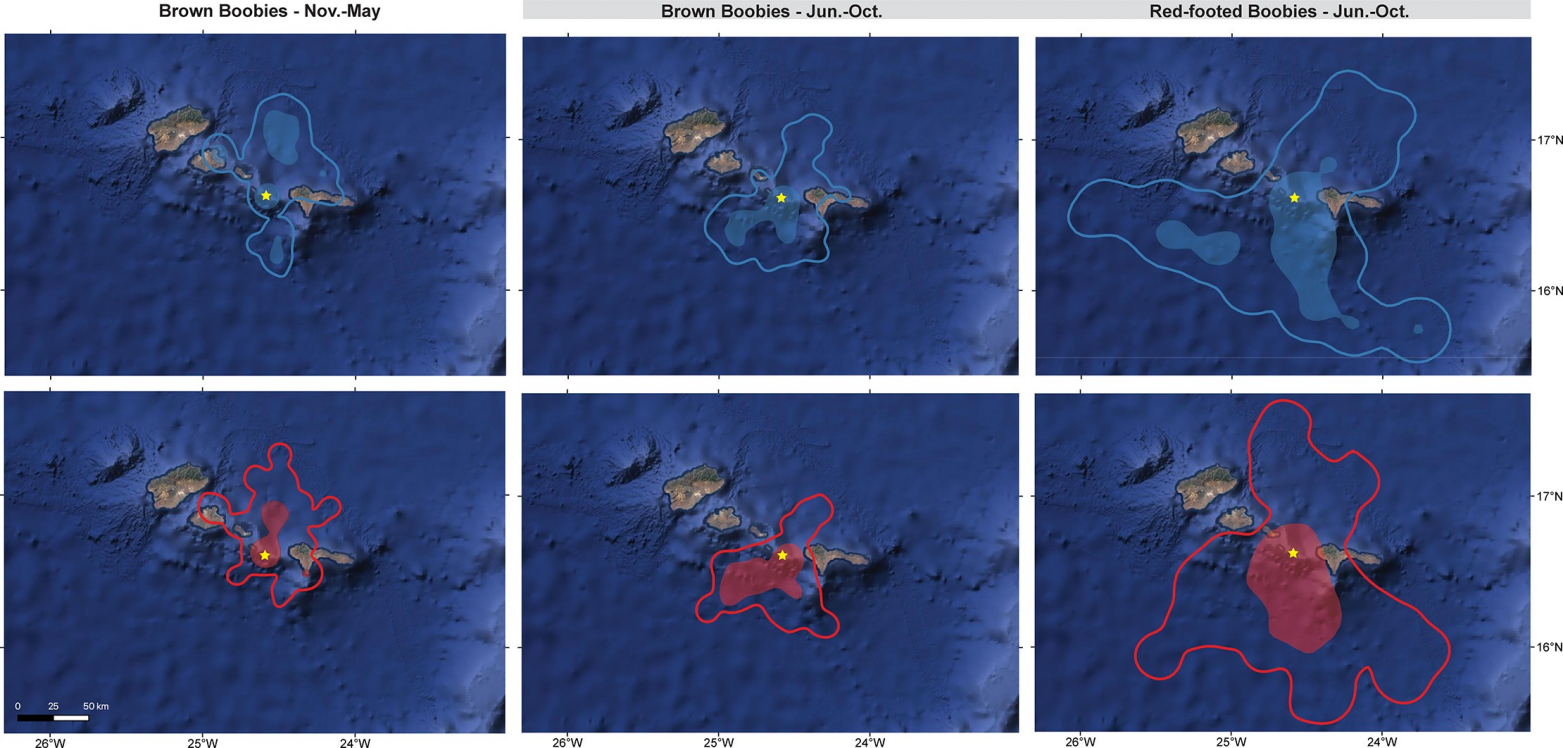
Kernel Utilization Distributions (UDs) of female (blue) and male (red) brown (BRBO) and red-footed (RFBO) boobies, tracked between November 2018 –October 2019 at Raso Islet (star), Cabo Verde. Bathymetric relief in the background (max. 4012m). Shaded area highlights the period when BRBO and RFBO co-occur in sympatry.

Our study took place in Raso Islet (16°36’40.63” N, 24°35’15.81” W) (Fig 1), located at ~16km from S. Nicolau Island, on the Cabo Verde archipelago. With 5.76 km^2^ of area, it is the biggest islet of the archipelago, located among the northern islands of “Barlavento” and belonging to the Integral Natural Reserve (Natural Reserves, Decree Law 3/II/03 of February 24) that also includes Santa Luzia Island and Branco Islet [44]. The islet holds two established colonies of BRBO, with ~289 breeding individuals, and one small population of RFBO, with ~133 non-breeding individuals occurring between June and October (Biosfera, unpublished data). The BRBO breeds throughout the year, but a peak in breeding numbers occurs in December-January.

Both sulid species (BRBO and RFBO) were captured using a long pole and net, and CatLog2 devices (Perthold Engineering LLC) were attached to their four central tail feathers. Devices were secured using Tesa® tape, programmed to record locations every 5 minutes, and retrieved 5–7 days after deployment (see Table 1 for details on sampling numbers). Each GPS weighed 19 g, well below the 3% body weight (BRBO weight: 1246 ± 227 g; weight: RFBO weight: 1041 ± 178 g) threshold recommended to avoid causing negative effects on the bird foraging and feeding success [45]. GPS-loggers’ deployment and retrieval were carried out after dark at the colony sites, and biometric data (wing-length, tarsus-width, body mass) were collected during logger deployment. At logger retrieval, data on body mass was collected once again to assess individual body condition, and blood (1ml) was drawn from the wing´s brachial vein and used for stable isotope analysis. Bird handling time did not exceed 10 minutes to avoid added stress to the animal. Body Condition Index (BCI) was obtained from the residuals of the linear regression of body weight on wing length–a measure of structural size [46]. BCI is, therefore, a measure of mass corrected for size and is considered an indicator of energetic reserves in seabirds [47]. Both species sex was identified using chest feather analysis with molecular markers, collected during field work [12,20]. Chick-rearing BRBO were tracked between September 2018 and August 2019, and non-breeding RFBO in September–October 2018 and June–August 2019, when this species inhabits Raso Islet (see further details on S1 Table).

**Table 1:** Summary statistics (mean±SD, range in brackets) of trip characteristics, spatial distribution, trophic ecology and body condition of tracked brown (BRBO) and red-footed (RFBO) boobies from Raso Islet, Cabo Verde.

Species	Brown booby				Red-footed booby	
Season	Nov–May		June–October		June–October	
Sex	female	male	female	male	female	male
N tracks [N birds]	136 [22]	189 [29]	175 [17]	207 [48]	36 [11]	98 [26]
Mass (kg)	1.38 ± 0.12 (1.26–1.54)	1.19 ± 0.17 (1.00–1.42)	1.44 ± 0.20 (1.28–1.69)	1.18 ± 0.16 (1.09–1.49)	1.02 ± 0.11 (0.97–1.20)	0.95 ± 0.25 (0.85–1.03)
Trip duration (h)	4.08 ± 0.11 (3.87–4.34)	3.12 ± 0.34 (2.76–3.98)	4.11 ± 0.42 (3.52–4.60)	5.19 ± 0.21 (4.76–5.47)	4.99 ± 0.53 (4.02–6.16)	5.70 ± 0.23 (5.09–5.88)
Max. dist. colony (km)	24.7 ± 3.21 (20.42–33.11)	31.17 ± 3.32 (21.67–34.65)	22.83 ± 3.93 (21.55–32.09)	42.60 ± 3.20 (35.52–49.33)	63.14 ± 4.2 (56.87–69.11)	55.73 ± 5.14 (51.12–68.52)
Prop. Travelling (%)	0.30 ± 0.32 (0.13–0.49)	0.31 ± 0.20 (0.13–0.69)	0.29 ± 0.20 (0.07–0.65)	0.39 ± 0.13 (0.11–0.55)	0.38 ± 0.10 (0.13–0.59)	0.39 ± 0.35 (0.09–0.75)
Prop. Foraging (%)	0.24 ± 0.15 (0.08–0.65)	0.27 ± 0.11 (0.13–0.59)	0.26 ± 0.24 (0.08–0.69)	0.29 ± 0.62 (0.09–0.76)	0.31 ± 0.10 (0.11–0.45)	0.32 ± 0.33 (0.08–0.65)
Prop. Resting (%)	0.22 ± 0.25 (0.12–0.54)	0.23 ± 0.35 (0.11–0.74)	0.19 ± 0.40 (0.07–0.78)	0.17 ± 0.51 (0.10–0.85)	0.29 ± 0.19 (0.09–0.49)	0.31 ± 0.13 (0.09–0.45)
Distal latitude (°)	17.14 ± 1.06 (15.69–17.98)	17.27 ± 0.82 (15.55–17.86)	16.04 ± 1.01 (15.67–17.06)	16.47 ± 0.82 (15.45–17.70)	16.44 ± 1.14 (14.68–17.53)	16.49 ± 0.94 (14.72–17.76)
Distal longitude (°)	-24.49 ± 1.26 (-25.78 –-23.94)	-24.26 ± 1.01 (-25.15 –-24.08)	-24.60 ± 0.50 (-25.09 –-24.01)	-24.32 ± 0.69 (-20.22 –-25.69)	-24.20 ± 0.91 (-25.09 –-21.77)	-24.24 ± 1.76 (-26.10 –-22.87)
Distal bearing (°)	45.92 ± 2.98 (13.21–59.43)	56.60 ± 1.83 (49.43–58.04)	181.36 ± 1.72 (179.31–184.23)	146.23 ± 2.19 (141.65–149.23)	135.3 ± 2.87 (131.23–141.99)	137.46 ± 2.01 (132.01–139.62)
Plasma *δ* ^13^C (‰)	-17.51 ± 0.09 (-17.94 –-16.35)	-17.4 ± 0.12 (-18.19 –-16.10)	-16.59 ± 0.17 (-17.42 –-14.71)	-16.31 ± 0.10 (-17.52 –-14.63)	-16.25 ± 0.13 (-16.94 –-15.02)	-16.29 ± 0.13 (-17.66 –-13.70)
Plasma *δ* ^15^N (‰)	11.70 ± 0.10 (10.61–12.87)	11.5 ± 0.14 (10.50–12.53)	10.93 ± 0.08 (10.03–12.13)	11.03 ± 0.09 (10.23–11.97)	11.77 ± 0.11 (10.49–13.07)	11.37 ± 0.06 (10.04–12.44)
Body Condition Index (BCI)	0.23 ± 0.02 (0.17–0.28)	0.20 ± 0.01 (0.18–0.25)	0.20 ± 0.03 (0.17–0.27)	0.16 ± 0.04 (0.08–0.24)	0.12 ± 0.05 (0.04–0.26)	0.13 ± 0.02 (0.11–0.22)

### Characterization of at-sea behaviours

To define individual behavioural modes from movement trajectories of foraging trips we used the *Expectation Maximisation binary Clustering* (EMbC) R package [48]. This is a robust non-supervised multi-variate clustering algorithm leading to meaningful local labelling of each GPS location that can be easily linked to biological interpretations [49]. Two input variables (speed and turn angle) were used from successive individual locations to assign 4 behaviours by the *EMbC* algorithm: high velocity/low turning angle (HL), high velocity/high turning angle (HH), low velocity/low turning angle (LL) and low velocity/high turning angle (LH) [50]. Following [48], the behaviours were described as: (1) Resting, when locations showed low speed and low turn value (LL), indicating that the bird is resting at the sea surface; (2) Intensive foraging, representing Low speed while searching and High turn value (LH), when patches of prey are spotted; (3) Travelling, showing High speed and Low turn value (HL); and (4) Relocating, reflecting High turns at High speed (HH) as a change between restricted areas of intensive foraging. This technique has been previously used to interpret ecologically meaningful behaviours from movement data in sulids [20,42,51].

### Habitat use

GPS locations labelled as ‘Intensive Foraging’ were used to generate Kernel Utilization Distributions (Kernel UD) estimates, considering the 50% kernel UD contours as the core foraging region (FR) and the 95% kernel UD contours as the home range (HR), using the *adehabitat* package in R (Calenge, 2006). The most appropriate smoothing parameter (*h*) was chosen via least squares cross–validation for the unsmoothed GPS data, and then applied as standard for the other datasets. Grid size was set at 0.08° to match the grid of environmental predictors.

The extent of within-group foraging region (FR) and home-range (HR) overlap between (1) sexes of the same species and (2) between species when they co-occur (June-October) was estimated using the Bhattacharyya’s affinity index (BA) kernel UD overlap index. This is considered the most appropriate measure of overlapping space use and BA index range from 0 (no overlap) to 1 (identical UDs) [52]. We used a randomization technique (1000 randomizations of our dataset) to test the null hypothesis that there was no difference in the spatial distribution of different groups at test. If the null hypothesis is true, overlap between groups 50% and 95% kernel UDs should not differ significantly from that calculated if those groups were randomly assigned. *P*-values were determined by the proportion of random overlaps that were smaller than the observed overlap (see [53,54] for similar approaches).

### Environmental predictors

To map the environmental conditions of the foraging areas and the surrounding colony, we used Seafloor depth as our static variable (DEP, blended ETOPO1 product, 0.01° spatial resolution, m), and the following dynamic oceanographic variables: Chlorophyll-a concentration (CHLA, 0.04°, mgm^-3^), Sea Surface Temperature (SST, 0.04°, °C), Sea Surface Height (SSH, 0.08°, m), and Ocean Mixed Layer Thickness (OMLT, 0.08°, m). DEP was downloaded from http://ngdc.noaa.gov/mgg/global/global.html, SST and CHLA were extracted from http://oceancolor.gsfc.nasa.gov, while SSH and OMLT were downloaded from http://marine.copernicus.eu. Monthly averages composites were used for all dynamic predictors. Spatial gradients of all former variables (GDEP, GCHLA, GSST, GOMLT, GSSH) were obtained by estimating the proportional change (PC) within a surrounding 3 × 3 cell grid using a moving window as follows: PC = [(maximum value − minimum value) × 100/maximum value]. Gradients of dynamic variables are believed to be good indicators of oceanic fronts, while the GDEP was used as a proxy for slope. All oceanographic raster layers were rescaled at a spatial resolution of 0.08° prior to the habitat modelling exercise. Environmental predictors were processed with various functions within the *raster* package [55].

### Stable isotope analysis of bobbies’ blood samples

Blood samples were separated into red blood cells (RBC) and plasma by centrifugation at 12000 rpm for 5 min. Plasma has a half-life of about 3–5 days [56] (i.e. fast turnover rate), therefore it represents prey ingestion and trophic ecology of tracked individuals during the last trips before sampling [56]. We used plasma samples collected during field work to perform a stable isotope analysis (SIA) for *δ*^15^N (^15^N / ^14^N) and *δ*^13^C (^13^C / ^12^C). As *δ*^15^N values increase continuously (3–5‰) in marine food webs, conferring different isotopic signatures to different prey consumed by seabirds, the trophic level is recognized, while *δ*^13^C values increase at a slower step (~0.8‰), indicating birds feeding habitat [57–59]. There is a gradient of high to low values of δ^13^C from benthic and inshore to pelagic and offshore food webs, because the organic enrichment at the coast is gradually diluted towards the open ocean [60]. Each of the tracked birds was sampled upon return from a foraging trip, during logger retrieval.

In the laboratory, plasma samples were dried at 60°C for 24 h and homogenized. Successive rinses with a 2:1 chloroform-methanol solution was performed on the plasma for dilapidation [56]. Approximately 0.25–0.30 mg of each sample were weighed and encapsulated into tin foil cups for posterior processing. The carbon and nitrogen isotopic ratios of these were determined from continuous-flow isotope ratio mass spectrometry (CF-IRMS). Results were presented in the common *δ* notation as parts per mil (‰) and compared with values from the international standards Pee Dee Belemnite (PDB) for *δ*^13^C and atmospheric N2 for *δ*^15^N. Replicate measurements of internal laboratory standards (acetanilide) indicate precision < 0.2‰ for both *δ*^13^C and *δ*^15^N.

### Stable isotope analysis of prey samples

Prey species were collected in 2018 and 2019 in Cabo Verde waters, by local fishermen operating in the surroundings of Raso Islet or at local fish-markets, between June-October of each year, and therefore are contemporaneous with tracking of BRBO and RFBO. We selected the prey species that could be part of BRBO and RFBOs’ diet, according to the data available in the literature for these and other similar taxa [13,61–63]. All prey individuals were measured, weighted, and identified to the lowest possible taxonomic level. Fish species were identified with local guides, while squid specimens were identified using the lower beaks [64]. Approximately 0.5 gr of muscle of each prey were dried at 60°C for 24 h, submitted to successive rinses with a 2:1 chloroform-methanol solution for tissue’s dilapidation [56], and weighed and encapsulated into tin foil cups for SIA. Due to the high similarity between some prey isotopic signatures, we opted to pool them in three different groups: epipelagic fish, juvenile fish, and squid. Epipelagic fish was composed by fish species that inhabit in the epipelagic layers of the ocean (i.e., the upper 200 m): *Sardinella aurita* (N = 4), *Platybelone lovii* (N = 4), *Sardinella maderensis* (N = 4), *Selar crumenophthalmus* (N = 4), *Cephalopholis taeniops* (N = 4), *Cheilopogon* sp. (N = 4), *Sparisoma cretense* (N = 3), *Decapterus macarellus* (N = 4), *Myripristis jacobus* (N = 4; mean ± SD: *δ*^13^C = –16.98 ± 0.50; *δ*^15^N = 10.05± 0.78). Juvenile fish was composed by young fingerlings identified as epipelagic species (mean ± SD: *δ*^13^C = –18.47 ± 0.24, *δ*^15^N = 8.38 ± 0.43, N = 10). Squid comprised specimens of *Todarodes sagittatus* (N = 6) and *Callimachus rancureli* (N = 4; mean ± SD: *δ*^13^C = –17.02 ± 1.56, *δ*^15^N = 11.66 ± 2.18).

### Diet reconstruction using SI mixing models

Diet reconstruction of BRBO and RFBOs was carried out by combining predator and prey isotopic signatures, and computing Bayesian mixing models with functions within the *simmr* R package [65]. Two models were run separately for each season (Nov.-May and Jun.-Oct.), computing the predicted consumption of three main groups of prey (epipelagic fish, juvenile fish and squid) for males and females BRBO and RFBO. We used adult plasma isotopic signatures as our predator data and prey muscle isotopic signatures as our sources data [65,66], with no prior diet information being added to the model.

To the best of our knowledge there are no diet-tissue discrimination factor (DTDF) calculated for BRBO or RFBOs. These factors are often specific for taxon, tissue, and even diet-specific [66,67], making them one of the largest sources of bias for isotopic mixing model practices [66]. In this study we used the DTDF calculated for Atlantic puffins (*Fratercula arctica*) in a captive experiment developed by [67]; we chose the DTDF calculated for plasma *δ*^13^C and *δ*^15^N isotopic ratios (− 0.18‰ for *δ*^13^C and + 1.72‰ for *δ*^15^N) to compare directly with boobies’ plasma *δ*^13^C and *δ*^15^N values and minimize the error of the models. Although puffins and boobies have different ecology and live in different environments (temperate vs tropics), we believe that these DTDF are the most adjusted values for this study, considering other values available in the literature [68,69]. We considered a standard deviation of ± 1.0‰, in an attempt to further reduce the bias of DTDF, and account for possible differences between boobies and puffins [70].

### Data analysis

Generalized Linear Mixed Models (GLMMs) were built to test the effect of the independent variables (1) sex, (2) season (Jun.-Oct. or Nov.-May), and (3) their interaction on the mean values of (1) trip duration (h), (2) maximum distance to colony (m), proportion of time (3) travelling (4) in intensive foraging and (5) resting, distal (6) latitude and (7) longitude of forays, plasma (8) *δ*^13^C, (9) *δ*^15^N values and (10) Body Condition Index. (i.e., dependent variables). Differences between sex and season on the (11) bearing of foraging trips (i.e. circular data) were tested with circular ANOVAs through the *circular* package [71]. Dependent variables of GLMMs were transformed when they did not meet normality and homogeneity assumptions. Separate models were developed for BRBO and RFBOs aiming at simpler interpretations of their outputs (i.e., interactions with the variable sex would be difficult to interpret in complex models). Both (1) trip identity nested within bird identity and (2) month of sampling was included as a random effect to control for pseudo-replication and temporal variability in the productivity of the marine environment, respectively. This also helped to account for unbalanced sample sizes per month. Gaussian distribution of error terms and a log-link function were used in the modelling. Post-hoc multiple comparisons with Bonferroni correction were used to identify significant differences between categories of each independent variable. R packages used in the GLMMs were *lme4* [72] and *lmerTest* [73].

GLMMs we also used to calculate “individual-level repeatability” (*R*) in each of the former behavioural and foraging parameters (except for distal bearings), with the *rptR* R package [74,75] as:

$$R_{ind}=S^{2}{}_{A}/S^{2}{}_{ind}+S^{2}{}_{A}$$

where *S* ^2^_A_ is the inter-individual variance and *S* ^2^_ind_ the intra-individual variance of each individual over time. Thus, inter-individual and intra-individual variances reflect the variances between foraging trips between individuals and the same bird, respectively. Repeatability index (0–1) can be classified as low (*R*_ind_ < 0.25), moderate (0.25 > *R*_ind_ > 0.5) and high (*R*_ind_ > 0.5) [76,77]. For visual comparison, we also computed the “population-level repeatability”, which is a variation of the former formula: *R*_pop_ = *S* ^2^_A_ / *S* ^2^ + *S* ^2^_A_; where *S* ^2^_A_ is the inter-individual variance and *S* ^2^ is the global within-individual variance. We compared the adjusted repeatability (repeatability calculated in the full fixed- and random-effects models) with models including only the individual as random effect, without any fixed effect.

Generalized Additive Mixed Models (GAMMs) were used to test the effect of (1) sex and (2) all environmental predictors on the presence/ absence of foraging behaviour (grid cells with ‘intensive foraging’) by tracked BRBO during (model 1) Nov.-May and (model 2) Jun.-Oct. and RFBO during (model 3) Jun.-Oct. Sex was included as a categorical variable in the fixed effects part of each model using the argument “by” of the *mgcv* package [78], allowing smoothers to be built for each sex and environmental variable combination. Smoothers were fitted to each environmental variable using 3 to 5 regression splines with shrinkage to avoid overfitting [79,80]. Multi-collinearity among covariates was assessed using variance inflation factors (GVIFs, *AEDForecasting* library in R [81]), with variables exhibiting a value higher than 3 being excluded from the modelling exercise (S2 Table). GAMMs were built with a binomial family and logit link function for presence/absence (of ‘intensive foraging’) data. Bird identity and sampling month were included to control for individual and monthly environmental variability effects, respectively. We started running models including all the main effects, and the best candidate model was selected based on the second-order corrected Akaike’s information criterion (AIC). A difference of less than 2 was interpreted as competing models receiving a similar amount of support from the data [82]. As with GLMMs, the three separate models were developed to attain comprehensive interpretations of the outputs and easily compare the effect of different environmental predictors on foraging between study seasons (Nov.-May *vs*. Jun.-Oct.) and species (BRBO *vs*. RFBO).

To establish the isotopic niche between sexes, study species (BRBO *vs*. RFBO) and season (June-October *vs*. November-May) with the plasma stable isotope data, we used SIBER (Stable Isotope Bayesian Ellipses in R), which is based on a Bayesian framework that confers a robust comparison to be made among data sets concerning different sample sizes [83]. The area of the standard ellipse (SEA_C_, an ellipse having a 40% probability of containing a subsequently sampled datum) was adopted to compare isotopic values among groups (sexes, species and seasons) and their overlap in relation to the total niche width (both groups combined), and a Bayesian estimate of the standard ellipse and its area (SEA_B_) was used to test whether the isotopic niche of one group was narrower than that of other group [83]. We further compared other isotopic niche metrics between species, sex and seasons, such as (1) carbon range, the distance between max. and min. *δ*^13^C values; (2) nitrogen range, the distance between max. and min. *δ*^15^N values; (3) total area (TA), as the convex hull area encompassed by all values in a *δ*^13^C - *δ*^15^N bi-plot space; (4) mean distance to centroid (CD), as the average Euclidean distance of each isotopic value to the *δ*^13^C - *δ*^15^N centroid, where the centroid is the mean *δ*^13^C - *δ*^15^N value for all values in the food web; (5) mean nearest neighbour distance (NND), as the mean of the Euclidean distances to each value nearest neighbour in bi-plot space, and thus a measure of the overall density of ‘values packing’; (6) SD nearest neighbour distance (SDNND), as a measure of the evenness of ‘values packing’ in bi-plot space that is less influenced than NND by sample size.

All data are presented as mean ± SD, unless otherwise stated. Results were considered significant at P ≤ 0.05.

### Compliance with ethical standards

This project was authorized by the "National Directorate of the Environment" of Cabo Verde (DNA) to be carried out at Raso Islet, Desertas Islands Natural Reserve. All sampling procedures and/or experimental manipulations were reviewed and specifically approved as part of obtaining the field license. All tracking information included in this publication is stored at BirdLife International Seabird Tracking Database (www.seabirdtracking.org) under IDs 1441 and 1442. Supporting data are available from the corresponding author upon reasonable request.

## Results

### Living without conspecifics

**Fig 2:**
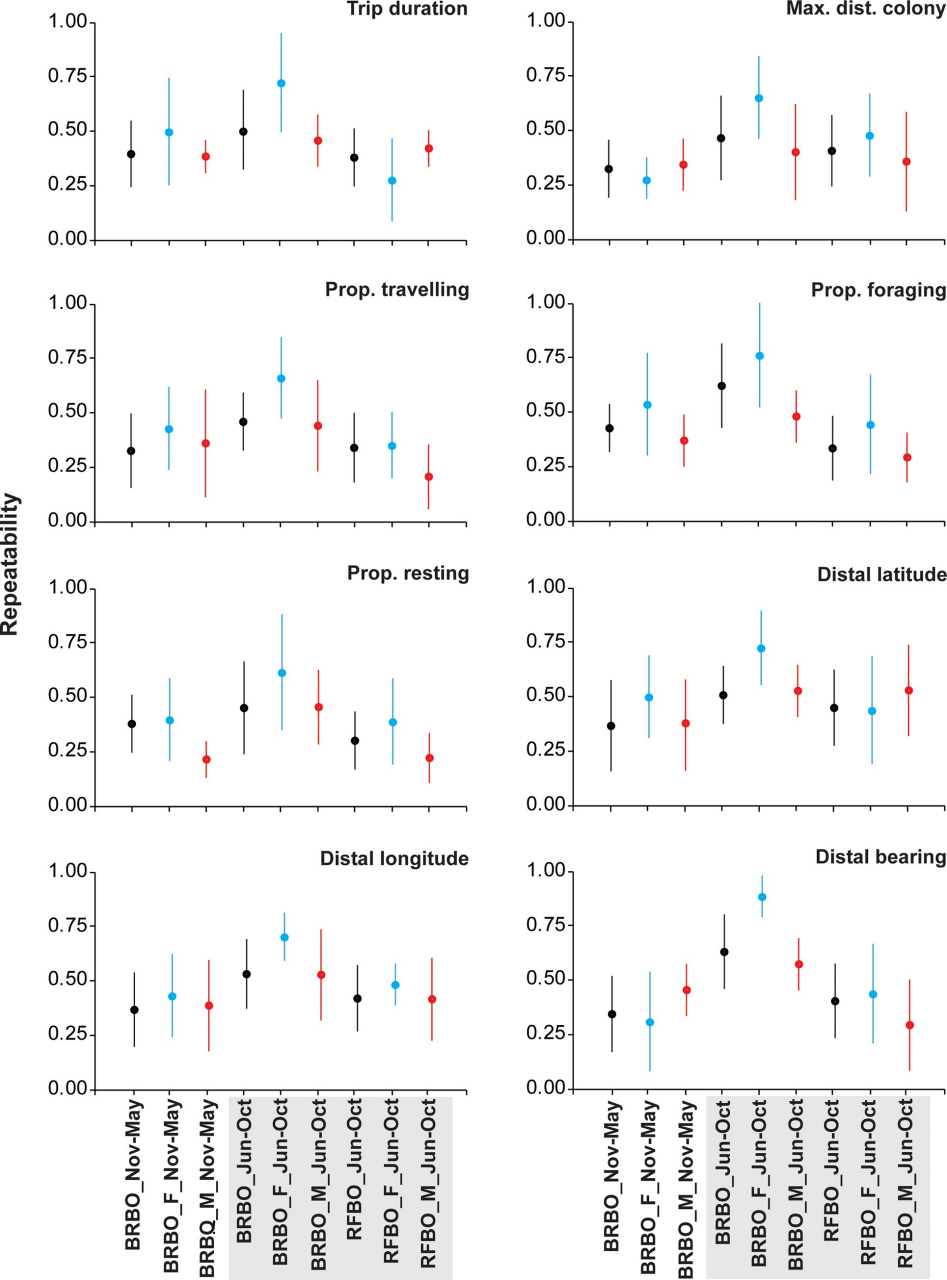
Population-level (*R*_pop_.; black) and individual-level (*R*_ind_) repeatability values (±SE) of behavioural parameters for female (blue) and male (red) brown (BRBO) and red-footed (RFBO) boobies. Shaded area highlights the period when BRBO and RFBO co-occur in sympatry at Raso Islet, Cabo Verde.

During Nov.-May, repeatability (*r*) of foraging behaviours and distribution was generally low (r < 0.25) to moderate (0.26< r < 0.5) for most of the parameters, and it was similar between male and female BRBO (Fig 2).

**Fig 3:**
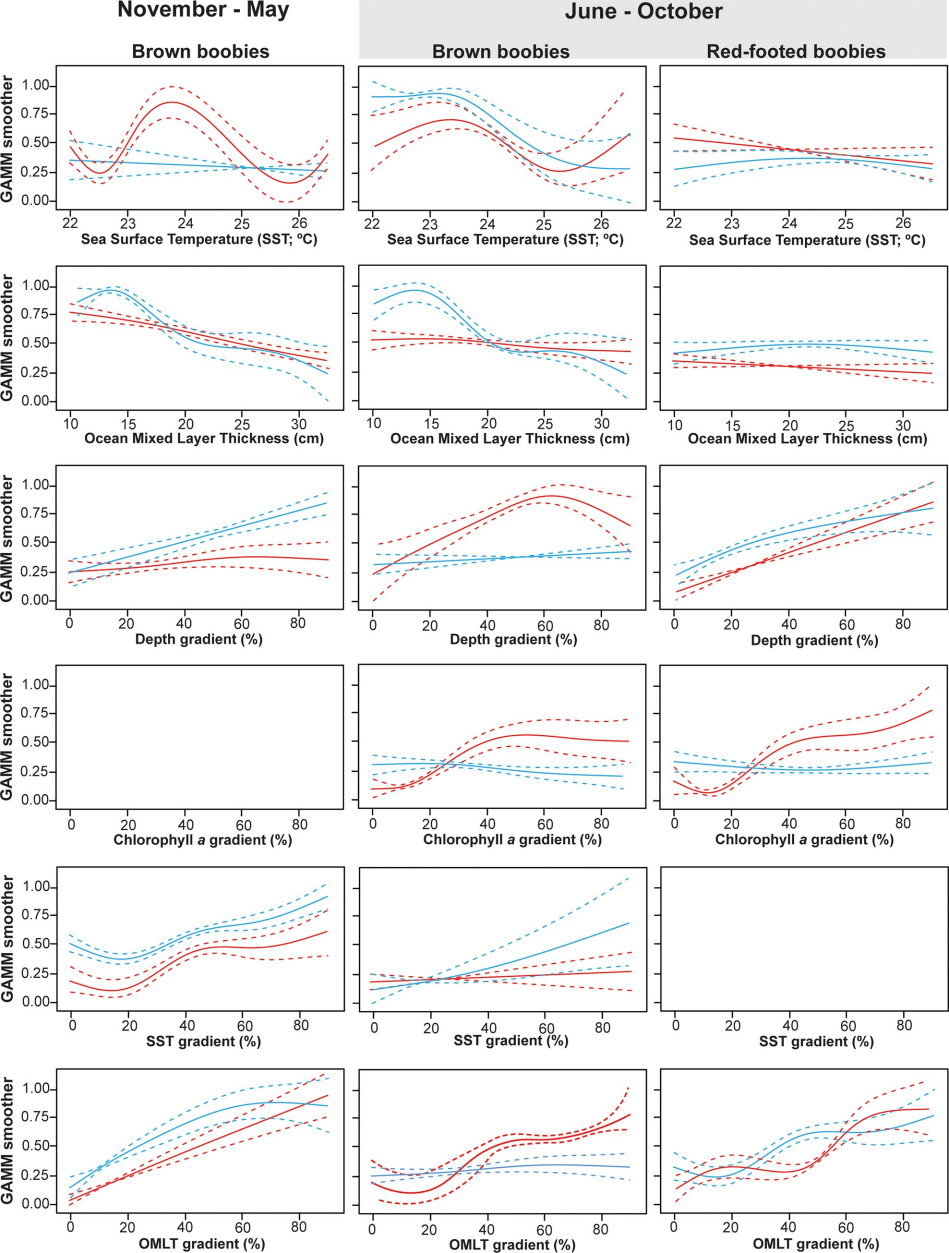
Response curves of the most important smooths resulting from generalized additive mixed models (GAMMs), explaining the foraging distribution of male (red) and female (blue) brown boobies (BRBO) and red-footed boobies (RFBO), during November–May and June–October. Shaded area highlights the period when BRBO and RFBO co-occur in sympatry at Raso Islet, Cabo Verde.

Overall, GAMMs showed a good predictive capacity, explaining 32.2%, 42.1% and 35.1% of the deviance in the probability of birds to switch between foraging and travelling behavioural modes (Table 2). Foraging probability of both female and male BRBO increased with decreasing ocean mixed layer thickness (OMLT) and increasing gradient in SST (GSST) and gradient in OMLT (GOMLT) (Fig 3).

**Table 2:** Generalized Additive Mixed Models (GAMMs) fitted to the probability to switch between foraging (1) and travelling (0) behavioural modes of tracked individual birds.

	Nov.-May			Jun.-Oct.					
	Brown boobies (BRBO)			Brown boobies (BRBO)			Red-footed boobies (RFBO)		
Term	edf	Chi.Sq	*P*	edf	Chi.Sq	*P*	edf	Chi.Sq	*P*
s(DEP): female	—	—	—	—	—	—	—	—	—
s(DEP): male	—	—	—	—	—	—	—	—	—
s(CHLA): female	0.34	0.43	0.87	—	—	—	0.44	0.34	0.21
s(CHLA): male	1.12	0.65	0.10	—	—	—	1.49	1.51	0.11
s(SST): female	0.01	0.01	0.41	1.36	2.42	**0.001**	0.01	0.01	0.79
s(SST): male	2.93	25.45	**< 0.001**	2.53	9.28	**0.001**	0.64	4.78	0.10
s(OMLT): female	1.57	2.74	**0.01**	2.02	4.00	**0.001**	0.11	0.19	0.39
s(OMLT): male	0.75	0.86	**0.02**	0.14	0.60	0.92	0.01	0.17	0.65
s(SSH): female	0.99	0.43	0.23	0.09	0.30	0.57	0.06	0.09	0.96
s(SSH): male	0.80	0.47	0.08	1.95	2.73	**0.01**	0.77	2.51	**0.04**
s(GDEP): female	0.83	1.45	**0.01**	0.04	0.23	0.34	0.98	13.27	**< 0.001**
s(GDEP): male	0.38	0.16	0.19	2.31	8.53	**0.001**	0.97	18.82	**< 0.001**
s(GCHLA): female	—	—	—	1.11	0.70	0.12	0.14	0.19	0.35
s(GCHLA): male	—	—	—	1.33	1.02	**0.05**	2.19	14.83	**0.001**
s(GSST): female	2.44	10.99	**< 0.001**	1.31	1.20	**0.03**	—	—	—
s(GSST): male	3.12	26.69	**< 0.001**	0.69	0.72	0.06	—	—	—
S(GOMLT): female	1.16	20.12	**< 0.001**	0.12	0.09	0.48	2.50	24.23	**< 0.001**
s(GOMLT): male	1.54	20.41	**< 0.001**	2.64	22.61	**< 0.001**	2.64	12.74	**0.001**
s(GSSH): female	—	—	—	0.17	0.05	0.50	0.15	0.08	0.50
s(GSSH): male	—	—	—	1.63	1.69	**0.01**	0.76	1.24	**0.04**

Three different GAMM models were built: (A) brown boobies during Nov.-May, (B) brown boobies during Jun.-Oct. and (C) red-footed boobies during Jun.-Oct. All evaluated models included individual identity and month as a random factors, to control for pseudo-replication and environmental variability, respectively; DEP–depth (m); CHLA–chlorophyll *a* concentration (mgm^-3^); SST—sea surface temperature (°C); OMLT–ocean mixed layer thickness (cm); SSH–sea surface height (m); GDEP–depth gradient (%); GCHLA–CHLA gradient (%); GSST–SST gradient (%); GOMLT–OMLT gradient (%); GSSH–SSH gradient (%).—Variables excluded after collinearity results. Significant results in bold.

Estimated diet composition of female and male BRBO was similar, with a higher estimated proportion of epipelagic and juvenile fish (including flying fish), and lower proportion of squids.

### Living in sympatry

During Jun.-Oct., male BRBO spent on average 1.42 hours more during each excursion, travelled ~17 km significantly further from their colony, and spent respectively 10.0% and 8.1% more time travelling and foraging, when compared to female BRBO. Plus, male BRBO exhibited 1.1 ‰ significantly lower plasma δ^13^C values and 7% lower body condition when compared to female BRBO (Fig 1; Tables 1 and 3A; S1 Fig).

**Table 3:** A. Generalized Linear Mixed Models (GLMMs) testing the effect of (A) the interaction between sex and season (November-May *vs*. June-October) and (B) the interaction between sex and species (brown boobies-BRBO *vs*. red-footed boobies-RFBO) on trip characteristics, spatial distribution, trophic ecology and body condition shown in Table 1.

**Model A**									
	**Sex**			**Season**			**Sex: Season**		
**Variables**	***F***_**3,703**_	***P***	**Effect**	***F***_**3,699**_	***P***	**Effect**	***F***_**3,699**_	***P***	**Effect**
N tracks [N birds]	—	—	—	—	—	—	—	—	—
Mass (kg)	—	—	—	—	—	—	—	—	—
Trip duration (h)	2.17	0.09	—	2.77	**0.05**	Nov-May < Jun-Oct	2.83	**0.04**	male, Jun-Oct > all others
Max. dist. colony (km)	5.48	**0.001**	females < males	2.69	**0.05**	Nov-May > Jun-Oct	2.71	**0.05**	male, Jun-Oct > all others
Prop. Travelling (%)	3.39	**0.02**	females < males	2.29	0.08	—	2.80	**0.04**	male, Jun-Oct > all others
Prop. Foraging (%)	3.81	**0.01**	females < males	3.90	**0.01**	Nov-May < Jun-Oct	3.30	**0.02**	male, Jun-Oct > all others
Prop. Resting (%)	1.59	0.19	—	2.81	**0.04**	Nov-May > Jun-Oct	1.65	0.18	—
Distal latitude (°)	3.69	**0.04**	females < males	3.09	**0.03**	Nov-May > Jun-Oct	1.19	0.33	—
Distal longitude (°)	6.01	**0.001**	females < males	1.76	0.18	—	1.44	0.27	—
Distal bearing (°)*	1.11	0.33	—	3.88	**0.01**	Nov-May < Jun-Oct	2.26	0.08	—
Plasma *δ* ^13^C (‰)	3.89	**0.01**	females < males	3.85	**0.01**	Nov-May < Jun-Oct	2.73	**0.05**	male, Jun-Oct < all others
Plasma *δ* ^15^N (‰)	1.37	0.25	—	2.75	**0.05**	Nov-May > Jun-Oct	1.95	0.12	—
Body Condition Index (BCI)	2.78	**0.04**	females > males	3.41	**0.02**	Nov-May > Jun-Oct	2.62	**0.05**	male, Jun-Oct < all others
**Model B**									
	**Sex**			**Species**			**Sex: Species**		
**Variables**	***F***_**3,507**_	***P***	**Effect**	***F***_**3,699**_	***P***	**Effect**	***F***_**3,699**_	***P***	**Effect**
N tracks [N birds]	—	—	—	—	—	—	—	—	—
Mass (kg)	—	—	—	—	—	—	—	—	—
Trip duration (h)	3.33	**0.02**	females > males	3.82	**0.01**	BRBO < RFBO	2.63	**0.05**	female, BRBO < all others
Max. dist. colony (km)	8.02	**< 0.001**	females < males	5.60	**0.001**	BRBO < RFBO	7.18	**< 0.001**	female, BRBO < all others
Prop. Travelling (%)	2.19	0.09	—	1.85	0.14	—	3.09	**0.03**	female, BRBO < all others
Prop. Foraging (%)	1.78	0.15	—	3.35	**0.02**	BRBO < RFBO	5.61	**0.001**	female, BRBO < all others
Prop. Resting (%)	1.68	0.17	—	2.83	**0.04**	BRBO < RFBO	2.37	0.07	—
Distal latitude (°)	3.87	**0.01**	females < males	3.67	**00.04**	BRBO < RFBO	2.88	**0.03**	female, BRBO < all others
Distal longitude (°)	3.01	**0.01**	females < males	1.98	0.11	—	3.99	**0.01**	female, BRBO < all others
Distal bearing (°)*	1.52	0.21	—	5.59	**0.001**	BRBO > RFBO	5.59	**0.001**	female, BRBO < all others
Plasma *δ* ^13^C (‰)	2.10	0.10	—	2.69	**0.05**	BRBO < RFBO	3.00	**0.03**	female, BRBO < all others
Plasma *δ* ^15^N (‰)	1.15	0.33	—	3.14	**0.03**	BRBO < RFBO	1.91	0.13	—
Body Condition Index (BCI)	1.79	0.13	—	2.79	**0.04**	BRBO > RFBO	2.81	**0.04**	female, BRBO > all others

Both (1) trip ID nested within bird ID and (2) Month of sampling were set as random effects to control for pseudo-replication and temporal variability on the environmental proxies of productivity, respectively. Significant results in bold. Effect was evaluated with Post-hoc multiple comparisons with Bonferroni correction.

* Differences between means of circular data variables were analysed with circular ANOVAs.

The observed foraging overlap between sexes and the overlap of females’ foraging distribution between seasons was similar to permuted overlap (Table 4). Yet, observed male-male foraging overlap between seasons and female-male foraging overlap during Jun.-Oct. was significantly lower than randomly expected (Table 4).

**Table 4:** Observed and randomized overlap (Bhattacharyya’s Affinity) at the 50% and 95% Kernel utilization distributions (UDs) between (A) female and male brown boobies during November–May and June–October and (B) female and male brown (BRBO) and red-footed boobies (RFBO) during June–October.

	50% UD			95% UD		
Comparison	Observed overlap	Permuted overlap (mean ± SD)	*P*	Observed overlap	Permuted overlap (mean ± SD)	*P*
**(A) Between sexes and seasons**						
female BRBO Nov.-May *vs*. male BRBO Nov.-May	0.38	0.35 ± 0.04	0.69	0.71	0.75 ± 0.03	0.56
female BRBO Nov.-May *vs*. female BRBO Jun.-Oct	0.39	0.41 ± 0.05	0.61	0.68	0.70 ± 0.05	0.59
male BRBO Nov.-May *vs*. male BRBO Jun.-Oct	0.25	0.40 ± 0.03	**0.02**	0.51	0.79 ± 0.05	**0.01**
female BRBO Jun.-Oct. *vs*. male BRBO Jun.-Oct.	0.27	0.30 ± 0.05	0.06	0.49	0.76 ± 0.03	**0.04**
**(B) Between sexes and study species**						
female BRBO *vs*. male BRBO	0.29	0.36 ± 0.06	0.06	0.50	0.69 ± 0.04	**0.04**
female BRBO *vs*. female RFBO	0.22	0.38 ± 0.04	**0.02**	0.46	0.64 ± 0.06	**0.01**
female BRBO *vs*. male RFBO	0.26	0.41 ± 0.05	**0.02**	0.43	0.61 ± 0.05	**0.01**
female RFBO *vs*. male RFBO	0.42	0.33 ± 0.06	**0.05**	0.44	0.59 ± 0.04	**0.03**

*P* represents the proportion of randomized overlaps that were smaller than the observed overlap. Significant differences are shown in bold.

During this period, when both BRBO and RFBO inhabit Raso Islet, female BRBO spent 1.2 hours less during each excursion, travelled ~29 km closer to the colony, and spent respectively 9.3% and 6.1% less time travelling and foraging, when compared to male BRBO and male and female RFBO. Moreover, female BRBO foraged over significantly lower latitude, longitudes, and distal bearings. Plus, they also exhibited 0.5‰ lower plasma δ^13^C values, though a 4% higher body condition when compared to male BRBO and male and female RFBO (Fig 1; Tables 1 and 3B; S1 Fig). The observed foraging overlap between sexes and between species was generally significantly lower during Jun.-Oct. than permuted overlap (Table 4).

Repeatability of female BRBO generally increased significantly to high (r > 0.6), while male BRBO and both sexes of RFBO remained on low to moderate repeatability of foraging behaviours and distribution (Fig 2).

In terms of habitat preference, foraging probability in female BRBO increased with decreasing SST and OMLT and increasing GOMLT. However, male BRBO and male and female RFBO generally increased foraging probability with increasing sea surface height (SSH), and gradients in seafloor depth (GDEP), chlorophyll *a* concentration (GCHLA), OMLT (GOMLT) and SST (GSST) (Table 4, Fig 3).

**Fig 4:**
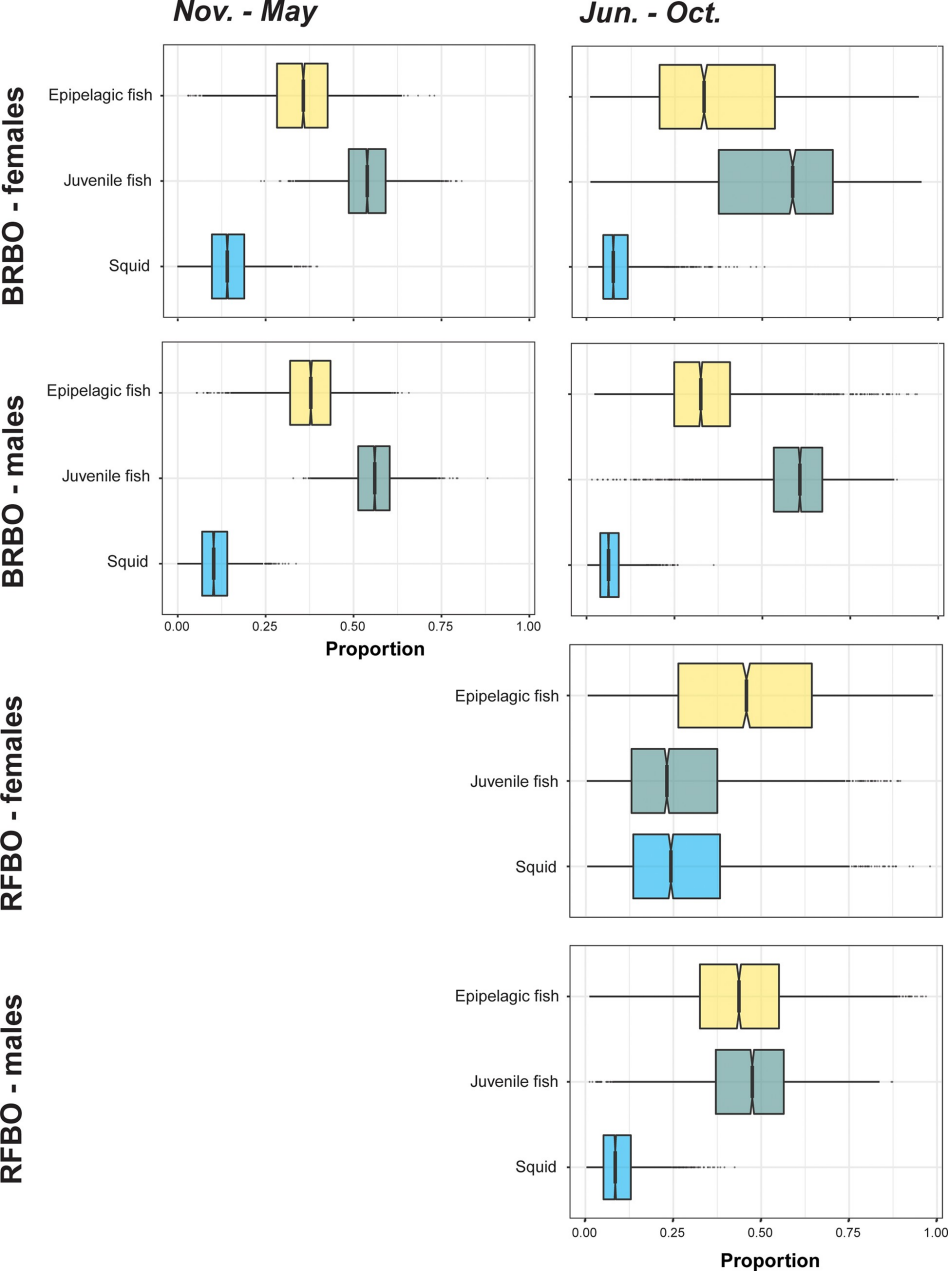
Estimated proportions of main prey items in the diet of male and female brown boobies (BRBO) and red-footed boobies (RFBO) from Raso Islet, Cabo Verde during a phase when only brown boobies occur on the colony (November-May; left panels) and when the two species co-occur in sympatry (June-October; right panels). Epipelagic fish–*Sardinella aurita*, *Platybelone lovii*, *Sardinella maderensis*, *Selar crumenophthalmus*, *Cephalopholis taeniops*, *Cheilopogon* sp., *Sparisoma cretense*, *Decapterus macarellus*, *Myripristis jacobu*; Juvenile fish–young fingerlings of epipelagic species; Squid–specimens from the Ommastrephidae and Onychoteuthidae families.

Models suggest that female BRBO did increase the consumption of epipelagic and juvenile fish, and similar patterns were found for female RFBO. On the other hand, males of both species fed on a slightly lower proportion of these food items when compared to females. The models also showed that while feeding on high proportion of epipelagic fish, female RFBO would rely also on comparable higher proportion of squid (Fig 4).”

Both male and female BRBO enlarged their isotopic niches from Nov.-May to Jun.-Oct. Female RFBO exhibited the larger SEA_C_ (S3 Table) and lower isotopic niche overlap in relation to male and female BRBO, but higher overlap with male RFBO isotopic niche (S2 Fig, S4 Table).

## Discussion

### Living without conspecifics

During Nov.-May, spatial segregation was very low between sexes of BRBO, a period when this is the only booby species occurring on Raso islet. Our results showed similar habitat use and repeatability of behavioural foraging parameters and distribution between sexes, as well as diet patterns, in line with Brown Boobies from islands in the Gulf of California [84]. There, colonies of BRBO are much larger (2,400–6,000 individuals) than those at Raso (~289 individuals) (S5 Table), but still no significant differences were observed in sexual foraging patterns in either place, meaning that density-dependent competition is not likely to drive sexual segregation.

Differences in sex-specific foraging patterns have mainly been related to marine productivity in the surrounding colonies [15,23], allowing for flexible parental investment to occur during breeding [15]. In our case, neither differential parental roles [85], niche specialization associated with sexual dimorphism and competitive exclusion [86], or nutritional requirements [87] affected foraging behaviour between sexes [84], because no segregation was observed. Other related species, such as Masked boobies have also shown similar foraging trip parameters, suggesting that local oceanic conditions and food distribution and availability are important factors leading to sexual segregation. Thus, these studies, imply that competition may not be an option when species are feeding on ephemeral prey, consequently compelling sexes to share food resources and foraging areas [21,42]. Therefore, as tropical marine regions such as the Cabo Verde Islands, are associated with oligotrophic waters and patchily distributed fish [9], this equal sharing of resources and habitats within species can be expected here. Similar environmental conditions at Tromelin Island in the Western Indian Ocean [24] and Palmyra Attol in the Central Pacific Ocean [23], have also been suggested to partly explain the lack of inter-sex differences observed in masked and red-footed boobies (S5 Table). Additionally, we stress that during this period (Nov-May), a higher annual peak of breeding occurs for this species (Biosfera unpublished data), which could lead to a higher number of breeding pairs competing for food, but still, no differences were observed between sexes.

In terms of foraging habitat, as both sexes stayed relatively close to their colonies, certain physical features may occur around Raso Islet, allowing the occurrence of discrete productive habitats for subsurface and aerial predators year-round. Thus, both sexes of BRBO showed a preference for areas with decreasing OMLT and increasing gradients of SST and OMLT, during Nov.—May. The OMLT has often been related to the depth of the vertical temperature gradient (thermocline), located right beneath the Ocean Mixed Layer (OML), possibly working as a physical and biological barrier for fish species [88,89]. Gradients of SST have also been connected with ocean fronts, where prey is known to concentrate and made available to top-predators [39,90]. Stretching from Cabo Verde Islands to the coast of Cape Blanc (West Africa), an important frontal system occurs, mixing waters coming from the north and south hemispheres with different gradients of temperature, salinity, and velocity [91]. Even though, CHLA concentration was not identified by the models as a variable influencing foraging habitat preference during this period, it is important to note that at the adjacent African coast, the upwelling phenomena leads to a higher concentration of chlorophyll between 10-20°N [92], especially during winter and spring, stretching long filaments and eddies of chlorophyll-rich waters to the open ocean and to the Cabo Verde archipelago [93,94] (S3 Fig). This could provide better foraging opportunities closer to the colonies during this period.

### Living in sympatry

During this period (Jun.-Oct.), intra-specific segregation was observed within both sexes of BRBO, and inter-specific segregation occurred between two sulid species living in sympatry [15,23,24,43] in Raso Islet. Although several studies concerning BRBO (S5 Table) [13,15,16] and other sulid species [15,19] found that bigger females frequently perform longer and farther foraging trips than smaller males, our study showed the exact opposite, with female BRBO maintaining their previous foraging areas, whilst male BRBO and both sexes of RFBO looked for different foraging opportunities.

Many papers on sex-specific foraging behaviour have pointed out that males and females may adopt different foraging strategies in response to different factors, such as competition, prey and habitat preference, parental involvement, among others [12,13,15,53,95–97]. Differences found in sexual foraging at Raso are in line with those found [17] at Johnston Atoll, Central Pacific, where male BRBO foraged in far distant areas than females (S5 Table). In our study, when both booby species were living in sympatry on Raso, inter-sexual differences in foraging behaviour and distribution may be explained by divergent parental roles, as females tend to stay closer to the colony, showing high repeatability to foraging grounds, feeding more rapidly and quickly returning to the colonies to feed their offspring. Several studies of boobies [33] and other seabird species exhibiting high foraging site fidelity [98,99] were carried out in highly productive marine environments (temperate regions), where prey distribution is more predictable, while in tropical areas, it has been pointed out that the presence of bathymetric features may be an important variable in increasing site fidelity [34]. Social cues, by gathering information from previous foraging trips [100], information transfer at sea [18,25,101] and at the colony [26], have also been suggested to increase site fidelity, however all were from studies in temperate areas. As females simply kept using the same areas, increasing repeatability of foraging parameters compared to the first period, and no important bathymetric features seem to be present or influencing utilized areas, this further suggests that parental involvement during this period, should be a factor explaining sexual segregation [13,15,16]. As all tracked BRBO were rearing chicks, feeding on higher proportions of epipelagic and juvenile fish could explain female´s better body condition, and also mean high energetic meals delivered to the offspring, further supporting this conclusion and explaining site fidelity [97,102].

Males on the other hand, would probably invest less on the chick, preferring to travel farther and using different foraging habitats, while taking advantage of winds to minimize flying costs [103] and increase flying efficiency (flight speed, foraging range and flapping frequency) [15,17]. Intuitively, this could also indicate a male habitat preference [53] during a period of sympatry. Lower body condition in males could indicate that they have a harder time foraging, and therefore invest less in chick provisioning [97]. Additionally, low repeatability to foraging grounds could reflect prey-patch unpredictability in the oligotrophic waters of Cabo Verde [6].

[13] also pointed out a possible division of labour between sexes at Reine Island (Great Barrier Reef, Australia), where males stayed longer at the nest and females travelled longer to bring food to the growing chick and returned later at sunset to avoid klepto-parasitism by frigate birds [13]. This suggests that inter-specific competition could also play a role in affecting intra-specific segregation of sulids living in sympatry with other species. In our study, because other sulid species moves to the Islet, competition pressure may contribute to sexual segregation. Thus, we also suggest that the presence of other seabird species, such as the Cape Verde shearwater (breeding from June-October) [104] and the Cape Verde little shearwater (prospecting) (Biosfera unpublished data), could also affect the foraging behaviour of BRBO. To further support this theory, at Raine island, BRBO were shown to exhibit intra-specific niche partitioning during breeding peaks [13], while during a period of low breeding effort no intra-specific niche segregation occurred [43]. This clearly demonstrates that BRBO can modify their intra-specific foraging behaviour to counterbalance possible competition for feeding resources. In our study, although the breeding peak of BRBO occurs between December-February, it is undeniable that the overall seabird breeding peak at Raso occurs during the summer period (Jun-Oct) [41,104–107]. Adding that a second colony of breeding Cape Verde shearwater and other species occur at less than 6.5 km away, in Branco islet [41,105], is enough to suppose that competition is a possibility between species.

In summary, regardless of whether female BRBO travelled longer than males, or stayed closer to the colonies, all studies agreed that differential breeding involvement, division of labour or sex role partitioning, in addition to competition, could have a double effect on sexual segregation [13,15,16]. Contrary to [17], body size does not appear to be the factor explaining intra-specific segregation, because seasonal variations in foraging parameters and isotopic niches were observed for this species, implying foraging plasticity inferred by surrounding environmental characteristics affecting prey abundance and habitat preference, as well as inter-specific competition due to co-existence and breeding peaks.

Foraging patterns of RFBO were also generally similar between sexes, following with the former literature on this species [12,17,20,108]. The similitude of these patterns might have been driven by low intra-specific competition due to the small population size of RFBO inhabiting Raso Islet [23,28], or more likely driven by an unbalanced female:male sample size (N = 4:17) [43]. Nevertheless, a difference in size does not seem to affect intra-specific foraging distributions here, and the lack of breeding seems to be a reasonable explanation for the low intra-specific competition.

Concerning inter-specific differences, RFBO showed a tendency to fly further and longer compared to BRBO due to its pelagic nature and smaller size [23,43]. Similarly, at Palmyra Atoll, in the Central Pacific Ocean, RFBO undertook more pelagic trips than bigger-sized Masked Boobies, although in this case there was an established breeding colony with a much greater number of RFBO (1000–2500 pairs) (S5 Table) [23], which could imply a higher foraging effort. This higher effort was also observed in larger colonies of Cape gannets (17,000–70,000 pairs) [31] and Masked boobies (4,600 individuals) [32], as competition is expected to be higher. On the other hand, at Tromelin Island (Indian Ocean), where colonies of both species are much smaller and fairly similar in numbers (RFBO = 180 pairs; MB = 250 pairs), Masked boobies travelled further than RFBO [24], which was not expected, as the larger size of Masked boobies would probably confer them the ability to outcompete the smaller RFBO from foraging areas in the colony surroundings [109]. In that case, environmental characteristics appeared to have a high influence on resource partitioning in an extreme oligotrophic environment [109].

In our study site, however, regardless of its tropical location and its less productive waters compared to the first period (S3 Fig), foraging bigger-sized female BRBOs seem to have relied on locations near the colony during both study periods, while smaller male BRBO and RFBO individuals foraged farther from the colony, in a more diverse array of areas, and exhibited lower repeatability in their foraging behaviour. From June to October, female BRBOs foraging probability increased with decreasing SST and OMLT, depicting good environmental conditions in areas closer to the colonies and a thinner mixed layer, which should allow prey fish to be closer to the surface and more accessible to predators [88,107,110–112]. Contrastingly, male BRBO and both sexes of RFBO preferred areas with higher gradients of SST (i.e. ocean fronts), chlorophyll *a* concentration (CHLA) (i.e. upwelling phenomena), and seafloor depth (i.e. steep locations), possibly along seamounts or shelf edges. Birds also showed a preference for areas with higher SSH values. High values of SSH usually depict the presence of Anticyclonic Eddies [113,114], which are also known to be associated with enhanced productivity (higher CHLA values), especially in tropical environments [115]. [116] showed the frequent occurrence of eddies inside and outside the Cabo Verde archipelago, which drive CHLA*-*rich waters from off West Africa and enhance CHLA within the archipelago. [117] also reported the formation of big anticyclonic eddies influenced by the Cabo Verde Frontal Zone (CVFZ). Similarly, studies in the Mozambique Channel (Europa Island) have shown the preference of the local seabird community (e.g. RFBOs) to forage in productive waters associated with the presence of mesoscale anticyclonic eddies, preferably around the edges of such oceanographic structures [108,118,119].

Both species explored the same prey species (squid, epipelagic and juvenile fish) which was expected, because sympatric tropical boobies studied in other locations exhibit similar dietary preferences (S5 Table) [8,21,23,118,120]. During winter and spring months, both sexes of BRBO had a similar expected diet, however, during the months of co-existence female BRBO showed a higher consumption of epipelagic and juvenile fish. This may be related to a greater availability of fish than squid in their foraging areas [121], and as suggested before, a possible selection of higher energetic-content prey to provision their chicks [24]. Flying fish have also been described to occur at the edges of rapidly rotating eddies [122], a type of habitat present in the waters of the Cabo Verde Islands, used by male BRBO and both sexes of RFBO. Nevertheless, results shown here on diet preferences evaluated from isotopic mixing models should be interpreted with caution, given the small sample size for each prey species. Ideally, future studies should sample potential prey all year-round, and increase sample size of each prey species to ascertain BRBO and RFBO dietary preferences, if possible, at the family- or species-levels.

Although intra-specific and inter-specific isotopic niches overlapped during the Jun.-Oct. period, there was a significant difference in *δ*^15^N values for females RFBO, as squid was expected to be more consumed by RFBO. This could be related to the higher abundance of squid during the summer months, main spawning and growth season [123], or even related to the use of different habitats [24]. Previous studies have connected the occurrence of squid (e.g. Ommastrephidae) with sea surface temperature and productivity connected to frontal zones [124,125] such as the one occurring in Cabo Verde [91]. A similar diet pattern was observed in two islands [12,24] of the Indian Ocean, where prey items of red-footed booby were mainly composed of squid, confirming the different trophic position of their prey. The fact that RFBO are not breeding may also be a contributing factor, because this species is not constrained by the need to feed chicks [126].

While BRBO expanded their isotopic niches across seasons, perhaps due to enhanced inter-species competition, there was a lower niche overlap of female RFBO in relation to BRBO, but higher when compared to that of male RFBO. This confirms the expected broader isotopic niche in a more generalist species like the RFBO during the non-breeding phase. Also, a high overlap in RFBO may indicate low intra-specific competition due to low individual numbers and non-breeding phase if reproductive duties are relaxed [43], or in this case inexistent, allowing adults to focus on their own nutritional needs.

### Conclusion

The current study provides the first view over the foraging ecology of a resident booby in Cabo Verde, with a second overview of changes when the pressure of another similar species is added to the study area. We conclude that divergent parental roles, environmental conditions, habitat preference and inter-specific competition could be mechanisms simultaneously underlying sexual segregation for BRBO during a period of co-existence. These results agree with the idea that BRBO boobies have a certain foraging ecology plasticity [13,43,84], capable of adapting to different circumstances of environmental conditions and competition. Foraging similarities in RFBO sexes, although confirmed in other studies, could possibly be related to the non-breeding phase or even biased sex sampling [43]. As such, inter-specific foraging differences, appear to be more affected by habitat preference and different breeding stages between species.

The results obtained here, are also pivotal for the identification of core foraging areas of both species, as an important input for future conservation plans to be applied within the Natural Reserve of Desertas Islands.

## Supporting information

S1 Fig Foraging tracks female (blue) and male (red) brown (BRBO) and red-footed (RFBO) boobies, tracked between November 2018 –October 2019 at Raso Islet (star), Cabo Verde. Bathymetric relief in the background (max. 4012m). Shaded area highlights the period when BRBO and RFBO co-occur in sympatry.
(DOCX)

S2 Fig Isotopic bivariate niche space of female (blue; filled line) and male (red; filled line) brown boobies (BRBO; *Sula leucogaster*) and female (blue; dashed line) and male (red; dashed line) red-footed boobies (RFBO; *Sula sula*). (DOCX)

S3 Fig Monthly average (±SD) sea surface temperature and chlorophyll *a* concentration 250km around Raso Islet between September 2018 –August 2019 (study period). (DOCX)

S1 Table Monthly summary of the number of trips and individual (between brackets) brown (BRBO) and red-footed (RFBO) boobies tracked in the current study. Shaded area highlights the period when BRBO and RFBO co-occur in sympatry.
(DOCX)

S2 Table Multi-collinearity among covariates selected for three Generalized Additive Mixed Models (GAMMs) assessed using variance inflation factors (GVIFs, *AEDForecasting* library in R. Highly colinear variables (VIF > 3) were removed prior to modeling. DEP–depth (m); CHLA–chlorophyll *a* concentration (mgm^-3^); SST–sea surface temperature (°C); OMLT–ocean mixed layer thickness (cm); SSH–sea surface height (m); GDEP–depth gradient (%); GCHLA–CHLA gradient (%); GSST–SST gradient (%); GOMLT–OMLT gradient (%); GSSH–SSH gradient (%).
(DOCX)

S3 Table Comparison of isotopic niche metrics between study species, sexes, and seasons (Nov.-May *vs*. Jun.-Oct.). Carbon range, the distance between max. and min. *δ*^13^C values; (2) nitrogen range, the distance between max. and min. *δ*^15^N values; (3) total area (TA), as the convex hull area encompassed by all values in a *δ*^13^C – *δ*^15^N bi-plot space; (4) standard ellipse area (SEA); (5) standard ellipse corrected for sample size (SEA_C_), depicting the area with 40% probability of containing a subsequently sampled datum; (6) mean distance to centroid (CD), as the average Euclidean distance of each isotopic value to the *δ*^13^C - *δ*^15^N centroid, where the centroid is the mean *δ*^13^C – *δ*^15^N value for all values in the food web; (7) mean nearest neighbour distance (NND), as the mean of the Euclidean distances to each value nearest neighbour in bi-plot space, and thus a measure of the overall density of ‘values packing’; (8) SD nearest neighbour distance (SDNND), as a measure of the evenness of ‘values packing’ in bi-plot space that is less influenced than NND by sample size.
(DOCX)

S4 Table Isotopic niche overlap (SEA_B_) between study species (brown booby; BRBO and red-footed booby; RFBO), sex and seasons (Nov.–May and Jun.–Oct.). (DOCX)

S5 Table Former studies reporting inter-sexual differences of brown (BB) and red-footed (RFB) boobies in their foraging distribution (foraging ≠), isotopic signatures (δ13C / δ15N), dietary preferences [FF–Flying fish (Exocetidae); FS–Flying squid (Ommastrephidae); SP–Small pelagic—anchovy, herring, sardines (Clupeidae)] and body mass (M = male; F = female). Shown are also other population-specific characteristics, namely ocean basin (P-Pacific, A-Atlantic, I-Indian), population size, Chlorophyll *a* concentration (CHLA) in the colony surroundings, mean breeding period and breeding stage (I–Incubation; CR–Chick-rearing) when the study was conducted. (1)–Differences during two periods of the year; (2)—F>M δ^13^C / F = M δ^15^N; (3) Females δ^15^N values higher than males; (4)–Females consumed more flying fish and males consumed equal proportions of fish and squid; (5)—Consumed mostly flying squid; (6)–Unspecified prey with high pelagic signature; (7)–Unspecified breeding phase.
(DOCX)
