# Supplementary material for: Year-round at-sea distribution and trophic resources partitioning between two sympatric Sulids in the tropical Atlantic
Source: PLoS One. 2021 Jun 21;16(6):e0253095. doi: 10.1371/journal.pone.0253095 (PMC8216530; doi:10.1371/journal.pone.0253095)
Supplement: S1 Fig — Bathymetric relief in the background (max. 4012m). Shaded area highlights the period when BRBO and RFBO co-occur in sympatry. (DOCX) [file pone.0253095.s001.docx]

**Electronic Supplementary Material**

**Year-round at-sea distribution and trophic resources partitioning between two sympatric Sulids in the tropical Atlantic**

Nathalie Almeida^1,2^, Jaime A. Ramos^1^, Isabel Rodrigues^2^, Ivo dos Santos^1^, Jorge M. Pereira^1^, Diana M. Matos^1^, Pedro M. Araújo^1,3^, Pedro Geraldes^4^, Tommy Melo^2^, Vitor H. Paiva^1^

*^1^ University of Coimbra, MARE – Marine and Environmental Sciences Centre, Department of Life Sciences, Calçada Martim de Freitas, 3000-456 Coimbra, Portugal;*

*^2^ Biosfera Cabo Verde, Rua de Moçambique 28, Mindelo, caixa postal 233, São Vicente, Cabo Verde;*

*^3^* *CIBIO/InBIO, Centro de Investigação em Biodiversidade e Recursos Genéticos, Campus Agrário de Vairão, Universidade do Porto, 4485-661 Vairão, Portugal.*

*^4^ SPEA - Sociedade Portuguesa para o Estudo das Aves, Av. Columbano Bordalo Pinheiro, 87, 3º Andar | 1070-062 Lisboa, Portugal.*


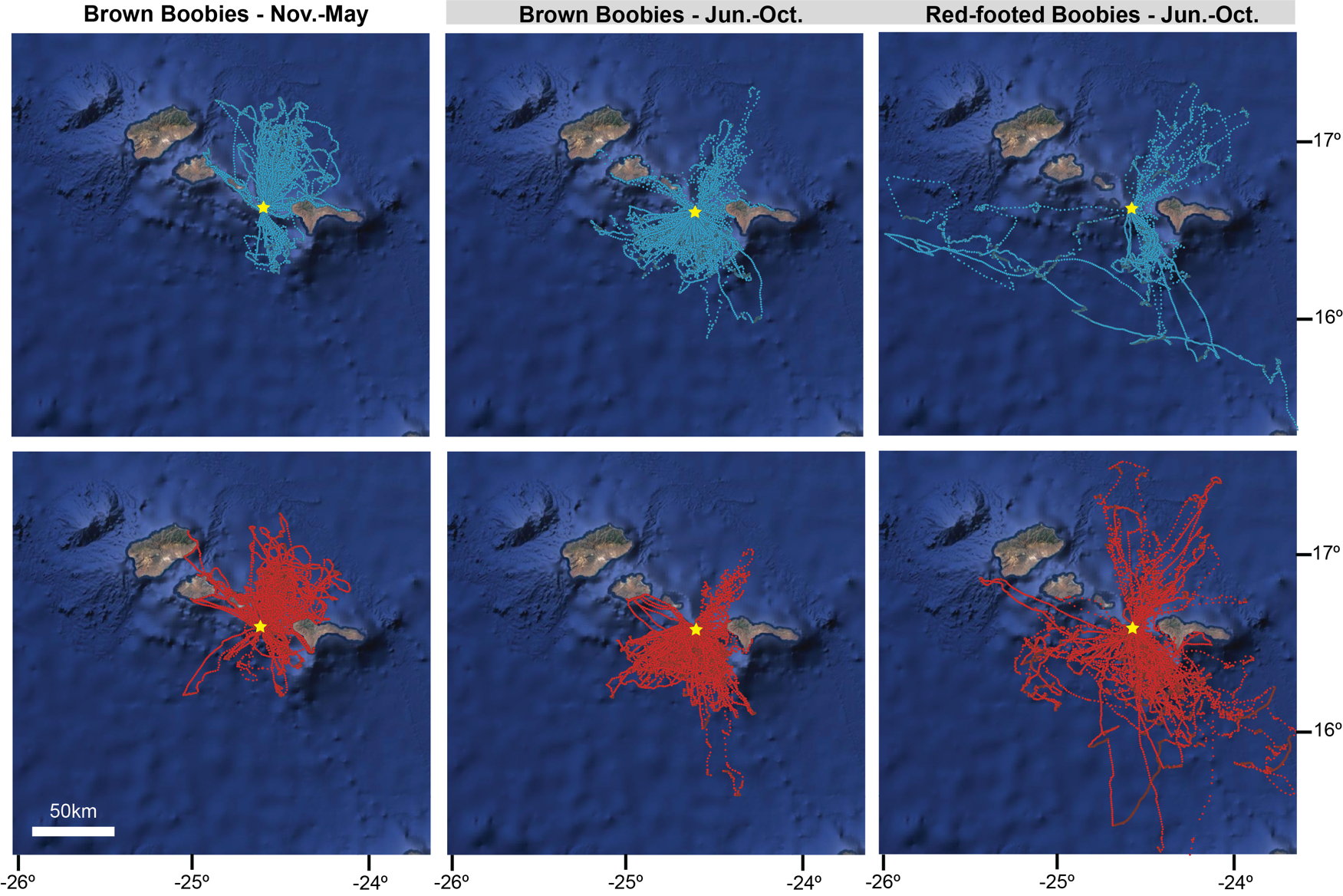


**S1 Fig. Foraging tracks female (blue) and male (red) brown (BRBO) and red-footed (RFBO) boobies, tracked between November 2018 – October 2019 at Raso Islet (star), Cabo Verde.** Bathymetric relief in the background (max. 4012m). Shaded area highlights the period when BRBO and RFBO co-occur in sympatry.
