# Supplementary material for: Year-round at-sea distribution and trophic resources partitioning between two sympatric Sulids in the tropical Atlantic
Source: PLoS One. 2021 Jun 21;16(6):e0253095. doi: 10.1371/journal.pone.0253095 (PMC8216530; doi:10.1371/journal.pone.0253095)
Supplement: S1 Table — Shaded area highlights the period when BRBO and RFBO co-occur in sympatry. (DOCX) [file pone.0253095.s004.docx]

**Electronic Supplementary Material**

**Year-round at-sea distribution and trophic resources partitioning between two sympatric Sulids in the tropical Atlantic**

Nathalie Almeida^1,2^, Jaime A. Ramos^1^, Isabel Rodrigues^2^, Ivo dos Santos^1^, Jorge M. Pereira^1^, Diana M. Matos^1^, Pedro M. Araújo^1,3^, Pedro Geraldes^4^, Tommy Melo^2^, Vitor H. Paiva^1^

*^1^ University of Coimbra, MARE – Marine and Environmental Sciences Centre, Department of Life Sciences, Calçada Martim de Freitas, 3000-456 Coimbra, Portugal;*

*^2^ Biosfera Cabo Verde, Rua de Moçambique 28, Mindelo, caixa postal 233, São Vicente, Cabo Verde;*

*^3^* *CIBIO/InBIO, Centro de Investigação em Biodiversidade e Recursos Genéticos, Campus Agrário de Vairão, Universidade do Porto, 4485-661 Vairão, Portugal.*

*^4^ SPEA - Sociedade Portuguesa para o Estudo das Aves, Av. Columbano Bordalo Pinheiro, 87, 3º Andar | 1070-062 Lisboa, Portugal.*

**S1 Table. Monthly summary of the number of trips and individual (between brackets) brown (BRBO) and red-footed (RFBO) boobies tracked in the current study.** Shaded area highlights the period when BRBO and RFBO co-occur in sympatry.

| **Year** | **Month** | **BRBO female** | **BRBO male** | **RFBO female** | **RFBO male** |
| --- | --- | --- | --- | --- | --- |
| 2018 | September | 38 (5) | 42 (5) | 17 (2) | 22 (7) |
| 2018 | October | 46 (4) | 25 (3) | 4 (2) | 35 (7) |
| 2018 | November | 50 (4) | 72 (6) | — | — |
| 2019 | February | 53 (9) | 57 (11) | — | — |
| 2019 | March | 5 (3) | 20 (3) | — | — |
| 2019 | April | 23 (3) | 27 (5) | — | — |
| 2019 | May | 5 (3) | 13 (4) | — | — |
| 2019 | June | 73 (9) | 89 (13) | 7 (2) | 25 (4) |
| 2019 | July | 13 (2) | 45 (21) | 4 (3) | 14 (6) |
| 2019 | August | 5 (3) | 6 (6) | 4 (2) | 2 (2) |
