# Supplementary material for: Year-round at-sea distribution and trophic resources partitioning between two sympatric Sulids in the tropical Atlantic
Source: PLoS One. 2021 Jun 21;16(6):e0253095. doi: 10.1371/journal.pone.0253095 (PMC8216530; doi:10.1371/journal.pone.0253095)
Supplement: S2 Table — Highly colinear variables (VIF > 3) were removed prior to modeling. DEP–depth (m); CHLA–chlorophyll a concentration (mgm-3); SST–sea surface temperature (°C); OMLT–ocean mixed layer thickness (cm); SSH–sea surface height (m); GDEP–depth gradient (%); GCHLA–CHLA gradient (%); GSST–SST gradient (%); GOMLT–OMLT gradient (%); GSSH–SSH gradient (%). (DOCX) [file pone.0253095.s005.docx]

**Electronic Supplementary Material**

**Year-round at-sea distribution and trophic resources partitioning between two sympatric Sulids in the tropical Atlantic**

Nathalie Almeida^1,2^, Jaime A. Ramos^1^, Isabel Rodrigues^2^, Ivo dos Santos^1^, Jorge M. Pereira^1^, Diana M. Matos^1^, Pedro M. Araújo^1,3^, Pedro Geraldes^4^, Tommy Melo^2^, Vitor H. Paiva^1^

*^1^ University of Coimbra, MARE – Marine and Environmental Sciences Centre, Department of Life Sciences, Calçada Martim de Freitas, 3000-456 Coimbra, Portugal;*

*^2^ Biosfera Cabo Verde, Rua de Moçambique 28, Mindelo, caixa postal 233, São Vicente, Cabo Verde;*

*^3^* *CIBIO/InBIO, Centro de Investigação em Biodiversidade e Recursos Genéticos, Campus Agrário de Vairão, Universidade do Porto, 4485-661 Vairão, Portugal.*

*^4^ SPEA - Sociedade Portuguesa para o Estudo das Aves, Av. Columbano Bordalo Pinheiro, 87, 3º Andar | 1070-062 Lisboa, Portugal.*

**S2 Table. Multi-collinearity among covariates selected for three Generalized Additive Mixed Models (GAMMs) assessed using variance inflation factors (GVIFs, *AEDForecasting* library in R.** Highly colinear variables (VIF > 3) were removed prior to modeling. DEP – depth (m); CHLA – chlorophyll a concentration (mgm^-3^); SST – sea surface temperature (°C); OMLT – ocean mixed layer thickness (cm); SSH – sea surface height (m); GDEP – depth gradient (%); GCHLA – CHLA gradient (%); GSST – SST gradient (%); GOMLT – OMLT gradient (%); GSSH – SSH gradient (%).

|  | **(A) brown boobies during Nov.-May** | | **(B) brown boobies during Jun.-Oct.** | | **(C) red-footed boobies during Jun.-Oct.** | |
| --- | --- | --- | --- | --- | --- | --- |
| **Env. variables** | **VIF value before selection** | **VIF value after selection** | **VIF value before selection** | **VIF value after selection** | **VIF value before selection** | **VIF value after selection** |
| DEP | 5.47 | — | 15.66 | — | 28.43 | — |
| CHLA | 2.14 | 2.06 | 5.47 | — | 2.55 | 2.19 |
| SST | 1.32 | 1.11 | 2.16 | 1.57 | 1.98 | 1.00 |
| OMLT | 1.05 | 1.00 | 2.00 | 1.24 | 1.34 | 1.06 |
| SSH | 2.00 | 1.78 | 1.84 | 1.22 | 2.39 | 2.22 |
| GDEP | 1.27 | 1.17 | 1.27 | 1.00 | 1.28 | 1.00 |
| GCHLA | 8.74 | — | 1.88 | 1.21 | 1.53 | 1.20 |
| GSST | 1.07 | 1.00 | 1.00 | 1.00 | 19.98 | — |
| GOMLT | 2.17 | 1.98 | 1.09 | 1.05 | 1.69 | 1.66 |
| GSSH | 9.69 | — | 2.47 | 2.31 | 2.47 | 2.40 |
