# Supplementary material for: Year-round at-sea distribution and trophic resources partitioning between two sympatric Sulids in the tropical Atlantic
Source: PLoS One. 2021 Jun 21;16(6):e0253095. doi: 10.1371/journal.pone.0253095 (PMC8216530; doi:10.1371/journal.pone.0253095)
Supplement: S3 Table — Carbon range, the distance between max. and min. δ13C values; (2) nitrogen range, the distance between max. and min. δ15N values; (3) total area (TA), as the convex hull area encompassed by all values in a δ13C – δ15N bi-plot space; (4) standard ellipse area (SEA); (5) standard ellipse corrected for sample size (SEAC), depicting the area with 40% probability of containing a subsequently sampled datum; (6) mean distance to centroid (CD), as the average Euclidean distance of each isotopic value to the δ13C - δ15N centroid, where the centroid is the mean δ13C – δ15N value for all values in the food web; (7) mean nearest neighbour distance (NND), as the mean of the Euclidean distances to each value nearest neighbour in bi-plot space, and thus a measure of the overall density of ‘values packing’; (8) SD nearest neighbour distance (SDNND), as a measure of the evenness of ‘values packing’ in bi-plot space that is less influenced than NND by sample size. (DOCX) [file pone.0253095.s006.docx]

**Electronic Supplementary Material**

**Year-round at-sea distribution and trophic resources partitioning between two sympatric Sulids in the tropical Atlantic**

Nathalie Almeida^1,2^, Jaime A. Ramos^1^, Isabel Rodrigues^2^, Ivo dos Santos^1^, Jorge M. Pereira^1^, Diana M. Matos^1^, Pedro M. Araújo^1,3^, Pedro Geraldes^4^, Tommy Melo^2^, Vitor H. Paiva^1^

*^1^ University of Coimbra, MARE – Marine and Environmental Sciences Centre, Department of Life Sciences, Calçada Martim de Freitas, 3000-456 Coimbra, Portugal;*

*^2^ Biosfera Cabo Verde, Rua de Moçambique 28, Mindelo, caixa postal 233, São Vicente, Cabo Verde;*

*^3^* *CIBIO/InBIO, Centro de Investigação em Biodiversidade e Recursos Genéticos, Campus Agrário de Vairão, Universidade do Porto, 4485-661 Vairão, Portugal.*

*^4^ SPEA - Sociedade Portuguesa para o Estudo das Aves, Av. Columbano Bordalo Pinheiro, 87, 3º Andar | 1070-062 Lisboa, Portugal.*

**S3 Table. Comparison of isotopic niche metrics between study species, sexes, and seasons (Nov.-May vs. Jun.-Oct.).** Carbon range, the distance between max. and min. *δ*^13^C values; (2) nitrogen range, the distance between max. and min. *δ*^15^N values; (3) total area (TA), as the convex hull area encompassed by all values in a *δ*^13^C – *δ*^15^N bi-plot space; (4) standard ellipse area (SEA); (5) standard ellipse corrected for sample size (SEA_C_), depicting the area with 40% probability of containing a subsequently sampled datum; (6) mean distance to centroid (CD), as the average Euclidean distance of each isotopic value to the *δ*^13^C - *δ*^15^N centroid, where the centroid is the mean *δ*^13^C – *δ*^15^N value for all values in the food web; (7) mean nearest neighbour distance (NND), as the mean of the Euclidean distances to each value nearest neighbour in bi-plot space, and thus a measure of the overall density of ‘values packing’; (8) SD nearest neighbour distance (SDNND), as a measure of the evenness of ‘values packing’ in bi-plot space that is less influenced than NND by sample size.

| **Species** | **Brown booby**  **(BRBO)** | | | | **Red-footed booby**  **(RFBO)** | |
| --- | --- | --- | --- | --- | --- | --- |
| **Study seasons** | **Nov. – May** | | **Jun. – Oct.** | | **Jun. – Oct.** | |
| **Sex** | **female** | **male** | **female** | **male** | **female** | **male** |
| **TA** | 2.72 | 3.69 | 3.50 | 3.68 | 2.65 | 5.94 |
| **SEA** | 1.09 | 1.11 | 1.76 | 1.53 | 3.28 | 2.28 |
| **SEA_C_** | 1.17 | 1.16 | 1.89 | 1.60 | 4.91 | 2.42 |
| **Carbon Range** | 1.83 | 2.23 | 2.66 | 2.79 | 1.86 | 3.98 |
| **Nitrogen Range** | 2.29 | 2.20 | 2.21 | 1.80 | 2.70 | 2.46 |
| **Mean Distance to Centroid (CD)** | 0.71 | 0.74 | 0.96 | 1.03 | 1.29 | 1.16 |
| **Mean Nearest Neighbour Distance (NND)** | 0.33 | 0.31 | 0.36 | 0.24 | 1.32 | 0.43 |
| **SD Nearest Neighbour Distance** | 0.28 | 0.27 | 0.17 | 0.16 | 0.72 | 0.32 |
