# Supplementary material for: Year-round at-sea distribution and trophic resources partitioning between two sympatric Sulids in the tropical Atlantic
Source: PLoS One. 2021 Jun 21;16(6):e0253095. doi: 10.1371/journal.pone.0253095 (PMC8216530; doi:10.1371/journal.pone.0253095)
Supplement: S4 Table — (DOCX) [file pone.0253095.s007.docx]

**Electronic Supplementary Material**

**Year-round at-sea distribution and trophic resources partitioning between two sympatric Sulids in the tropical Atlantic**

Nathalie Almeida^1,2^, Jaime A. Ramos^1^, Isabel Rodrigues^2^, Ivo dos Santos^1^, Jorge M. Pereira^1^, Diana M. Matos^1^, Pedro M. Araújo^1,3^, Pedro Geraldes^4^, Tommy Melo^2^, Vitor H. Paiva^1^

*^1^ University of Coimbra, MARE – Marine and Environmental Sciences Centre, Department of Life Sciences, Calçada Martim de Freitas, 3000-456 Coimbra, Portugal;*

*^2^ Biosfera Cabo Verde, Rua de Moçambique 28, Mindelo, caixa postal 233, São Vicente, Cabo Verde;*

*^3^* *CIBIO/InBIO, Centro de Investigação em Biodiversidade e Recursos Genéticos, Campus Agrário de Vairão, Universidade do Porto, 4485-661 Vairão, Portugal.*

*^4^ SPEA - Sociedade Portuguesa para o Estudo das Aves, Av. Columbano Bordalo Pinheiro, 87, 3º Andar | 1070-062 Lisboa, Portugal.*

**S4 Table. Isotopic niche overlap (SEA_B_) between study species (brown booby; BRBO and red-footed booby; RFBO), sex and seasons (Nov. – May and Jun. – Oct.).**

| **Comparisons** | **SEA_B_** | **Overlap** | **Prop 95%**  **overlap** |
| --- | --- | --- | --- |
| **Nov. – May** |  |  |  |
| **BRBO male *vs*. BRBO female** | 0.51 | 0.85 | 0.58 |
| **Jun. – Oct.** |  |  |  |
| **BRBO male *vs*. BRBO female** | 0.68 | 1.38 | 0.65 |
| **BRBO male *vs*. RFBO male** | 0.90 | 1.23 | 0.44 |
| **BRBO male *vs*. RFBO female** | 0.95 | 1.44 | 0.28 |
| **BRBO female *vs*. RFBO male** | 0.77 | 1.12 | 0.35 |
| **BRBO female *vs*. RFBO female** | 0.91 | 1.42 | 0.26 |
| **RFBO male *vs*. RFBO female** | 0.79 | 2.09 | 0.40 |
