# Supplementary material for: Year-round at-sea distribution and trophic resources partitioning between two sympatric Sulids in the tropical Atlantic
Source: PLoS One. 2021 Jun 21;16(6):e0253095. doi: 10.1371/journal.pone.0253095 (PMC8216530; doi:10.1371/journal.pone.0253095)
Supplement: S5 Table — Shown are also other population-specific characteristics, namely ocean basin (P-Pacific, A-Atlantic, I-Indian), population size, Chlorophyll a concentration (CHLA) in the colony surroundings, mean breeding period and breeding stage (I–Incubation; CR–Chick-rearing) when the study was conducted. (1)–Differences during two periods of the year; (2)—F>M δ13C / F = M δ15N; (3) Females δ15N values higher than males; (4)–Females consumed more flying fish and males consumed equal proportions of fish and squid; (5)—Consumed mostly flying squid; (6)–Unspecified prey with high pelagic signature; (7)–Unspecified breeding phase. (DOCX) [file pone.0253095.s008.docx]

**Electronic Supplementary Material**

**Year-round at-sea distribution and trophic resources partitioning between two sympatric Sulids in the tropical Atlantic**

Nathalie Almeida^1,2^, Jaime A. Ramos^1^, Isabel Rodrigues^2^, Ivo dos Santos^1^, Jorge M. Pereira^1^, Diana M. Matos^1^, Pedro M. Araújo^1,3^, Pedro Geraldes^4^, Tommy Melo^2^, Vitor H. Paiva^1^

*^1^ University of Coimbra, MARE – Marine and Environmental Sciences Centre, Department of Life Sciences, Calçada Martim de Freitas, 3000-456 Coimbra, Portugal;*

*^2^ Biosfera Cabo Verde, Rua de Moçambique 28, Mindelo, caixa postal 233, São Vicente, Cabo Verde;*

*^3^* *CIBIO/InBIO, Centro de Investigação em Biodiversidade e Recursos Genéticos, Campus Agrário de Vairão, Universidade do Porto, 4485-661 Vairão, Portugal.*

*^4^ SPEA - Sociedade Portuguesa para o Estudo das Aves, Av. Columbano Bordalo Pinheiro, 87, 3º Andar | 1070-062 Lisboa, Portugal.*

**S5 Table.** **Former studies reporting inter-sexual differences of brown (BB) and red-footed (RFB) boobies in their foraging distribution (foraging ≠), isotopic signatures (δ^13^C / δ^15^N), dietary preferences [FF – Flying fish (Exocetidae); FS – Flying squid (Ommastrephidae); SP – Small pelagic - anchovy, herring, sardines (Clupeidae)] and body mass (M=male; F=female).** Shown are also other population-specific characteristics, namely ocean basin (P-Pacific, A-Atlantic, I-Indian), population size, Chlorophyll *a* concentration (CHLA) in the colony surroundings, mean breeding period and breeding stage (I – Incubation; CR – Chick-rearing) when the study was conducted. (1) – Differences during two periods of the year; (2) - F>M δ^13^C / F=M δ^15^N; (3) Females δ^15^N values higher than males; (4) – Females consumed more flying fish and males consumed equal proportions of fish and squid; (5) - Consumed mostly flying squid; (6) – Unspecified prey with high pelagic signature; (7) – Unspecified breeding phase.

| Sps | Ocean | Colony | Population size | CHLA (mg m-3) | | Foraging ≠ | Isotopic Sig. | Diet | Weight (g) | Breeding | Breeding stage | | Publication |
| --- | --- | --- | --- | --- | --- | --- | --- | --- | --- | --- | --- | --- | --- |
| BB | P | Clipperton isl. | 15000-25000 ind. | | 0.2 [1] | F>M | - | - | - | Nov | I&CR | | Gilardi 1992 [2] |
|  | P | Johnston Atoll | 450 pairs | | - | F<M | - | - | M = 1077 ± 78  F = 1491 ± 76 | Mar | I | | Lewis et al 2005 [3] |
|  | P | Sanildefonso Isl. | 2000 pairs | | High | F>M | F=M | SP | M = 1194 ± 85  F = 1448 ± 86 | Mar | CR | | Weimerskirch et al 2009b [4] |
|  | P | Palmyra Atoll | ~400 pairs | | 0.14 | - | F>M | FF, SP, FS | - | Jul | I&CR | | Young et al 2010a [5] |
|  | A | Dog Isl. | 2462 ind. | | - | - | - | - | M = 1062  F = 1416 | Mar-Apr | CR | Soanes et al 2015 [6] | |
|  | I | Christmas Isl. | 6000 pairs | | - | - | F=M | - | - | Sep-Oct | I&CR | | Navarro et al 2014 [7] |
|  | P | San Jorge Isl. | 6000 ind. | | High | F=M | - | SP | - | Nov-May | I&CR | | Castillo-Guerrero 2016 [8] |
|  | P | Farallón Isl. | 2400 ind. | | High | F=M | - | SP | - | Jan-May | I&CR | | Castillo-Guerrero 2016 [8] |
|  | P | Raine Isl. | 2642 ind. | | - | F>M | - | FF, FS**^4^** | M = 1197 ± 65  F = 1430 ± 147 | Dec | CR | | Miller et al 2018 [9] |
|  | A | Raso Islet | ~289 ind. | | 0.15-0.38 | F=M / F<M**^1^** | F=M | FF, FS | 1246 ± 227 | Feb-Nov | CR | | This study |
| RFB | P | Johnston Atoll | 1400 pairs | | - | F=M | - | - | M = 1035 ± 70  F = 1182 ± 86 | Mar | I | | Lewis et al 2005 [3] |
|  | I | Europa Isl. | 2800-3800 pairs | | 0.165 | - | - | FF, FS | M = 780  F = 1050 | Aug-Sep | I&CR | | Weimerskirch et al 2005a [10] |
|  | I | Europa Isl. | 2800-3800 pairs | | 0.165 | - | - | FF, FS | M = 780  F = 1050 | Aug-Sep | I&CR | | Weimerskirch et al 2005b [11] |
|  | I | Europa Isl. | 2800-3800 pairs | | 0.165 | F(I) > M(I) | - | FF, FS**^5^** | M = 891 ± 64.4  F = 1020 ± 50.1 | Aug-Sep | I&CR | | Weimerskirch et al 2006 [12] |
|  | I | Europa Isl. | 9000 ind. | | - | F>M | F>M/F=M**^2^** | FF, FS | - | Aug-Sep | B – U**^7^** | | Cherel et al 2008 [13] |
|  | P | Palmyra Atoll | ~2500 pairs | | - | - | F=M | FF, SP, FS | - | Jul | I&CR | | Young, 2010a [5] |
|  | P | Palmyra Atoll | 1000-2500 pairs | | 0.1-0.2 | F=M | F=M | U**^6^** | M = 839 ± 44  F = 954 ± 41 | May-Nov | I&CR | | Young et al 2010b [14] |
|  | I | Tromelin Isl | 130-180 pairs | | 0.03-0.13 | F=M | - | FF, FS**^5^** | M = 920 ± 71.6  F = 1054 ± 46.2 | Dec-Jan | CR | | Kappes et al 2011 [15] |
|  | I | Europa Isl. | 2800-3800 pairs | | 0.153-0.154 | F=M | - | - | M = 800-900  F = 1000-1200 | Sep-Nov | I&CR | | Mendez et al 2016 [16] |
|  | I | Europa Isl. | 2800-3800 pairs | | 0.07 | F=M | - | - | - | Sep-Oct | I | | Mendez et al 2017 [17] |
|  | P | Walpole Isl. | 1000 pairs | | 0.07 | F=M | - | - | - | Sep | I | | Mendez et al 2017 [17] |
|  | P | Chesterfiled Isl. | 7200-7300 pairs | | 0.11 | F=M | - | - | - | May-Jun | I | | Mendez et al 2017 [17] |
|  | P | Genovesa Isl. | 140000 pairs | | > 0.15 | F=M | - | - | - | Nov | I | | Mendez et al 2017 [17] |
|  | I | Christmas Isl. | 12000 pairs | | > 0.15 | F=M | - | - | - | Jul-Aug | I | | Mendez et al 2017 [17] |
|  | A | Raso islet | ~133 ind. | | 0.15-0.38 | F=M | F>M**^3^** | FF, FS | 1041±178 | Jun-Oct | Non-breeding | | This study |

**References**

1. Weimerskirch H, Le Corre M, Bost CA. Foraging strategy of masked boobies from the largest colony in the world: Relationship to environmental conditions and fisheries. Mar Ecol Prog Ser. 2008;362: 291–302. doi:10.3354/meps07424

2. Gilardi JD. Sex-Specific Foraging Distributions of Brown Boobies in the Eastern Tropical Pacific. Colon Waterbirds. 1992;15: 148. doi:10.2307/1521367

3. Lewis S, Schreiber EA, Daunt F, Schenk GA, Orr K, Adams A, et al. Sex-specific foraging behaviour in tropical boobies: Does size matter? Ibis (Lond 1859). 2005;147: 408–414. doi:10.1111/j.1474-919x.2005.00428.x

4. Weimerskirch H, Shaffer SA, Tremblay Y, Costa DP, Gadenne H, Kato A, et al. Species- and sex-specific differences in foraging behaviour and foraging zones in blue-footed and brown boobies in the Gulf of California. Mar Ecol Prog Ser. 2009;391: 267–278. doi:10.3354/meps07981

5. Young HS, McCauley DJ, Dirzo R, Dunbar RB, Shaffer SA. Niche partitioning among and within sympatric tropical seabirds revealed by stable isotope analysis. Mar Ecol Prog Ser. 2010;416: 285–294. doi:10.3354/meps08756

6. Soanes LM, Bright JA, Bolton M, Millett J, Mukhida F, Green JA. Foraging behaviour of Brown Boobies Sula leucogaster in Anguilla, Lesser Antilles: Preliminary identification of at-sea distribution using a time-in-area approach. Bird Conserv Int. 2015;25: 87–96. doi:10.1017/S095927091400001X

7. Navarro J, Moreno R, Braun L, Sanpera C, Hennicke JC. Resource partitioning between incubating and chick-rearing brown boobies and red-tailed tropicbirds on Christmas Island. Zool Stud. 2014;53: 1–6. doi:10.1186/s40555-014-0027-1

8. Castillo-Guerrero JA, Lerma M, Mellink E, Suazo-Guillén E, Peñaloza-Padilla EA. Environmentally-Mediated Flexible Foraging Strategies in Brown Boobies in the Gulf of California. Ardea. 2016;104: 33–47. doi:10.5253/arde.v104i1.a3

9. Miller MGR, Silva FRO, Machovsky-Capuska GE, Congdon BC. Sexual segregation in tropical seabirds: drivers of sex-specific foraging in the Brown Booby Sula leucogaster. J Ornithol. 2018;159: 425–437. doi:10.1007/s10336-017-1512-1

10. Weimerskirch H, Corre M Le, Jaquemet S, Marsac F. Foraging strategy of a tropical seabird, the red- footed booby, in a dynamic marine environment.pdf. Mar Ecol Prog Ser. 2005;288: 251–261.

11. Dunn DC, Harrison AL, Curtice C, DeLand S, Donnelly B, Fujioka E, et al. The importance of migratory connectivity for global ocean policy. Proc R Soc B Biol Sci. 2019;286. doi:10.1098/rspb.2019.1472

12. Weimerskirch H, Corre M Le, Ropert-Coudert Y, Kato A, Marsac F. Sex-specific foraging behaviour in a seabird with reversed sexual dimorphism: The red-footed booby. Oecologia. 2006;146: 681–691. doi:10.1007/s00442-005-0226-x

13. Cherel Y, Corre M Le, Jaquemet S, Ménard F, Richard P, Weimerskirch H. Resource partitioning within a tropical seabird community: New information from stable isotopes. Mar Ecol Prog Ser. 2008;366: 281–291. doi:10.3354/meps07587

14. Young HS, Shaffer SA, McCauley DJ, Foley DG, Dirzo R, Block BA. Resource partitioning by species but not sex in sympatric boobies in the central Pacific Ocean. Mar Ecol Prog Ser. 2010;403: 291–301. doi:10.3354/meps08478

15. Kappes MA, Weimerskirch H, Pinaud D, Le Corre M. Variability of resource partitioning in sympatric tropical boobies. Mar Ecol Prog Ser. 2011;441: 281–294. doi:10.3354/meps09376

16. Mendez L, Cotté C, Prudor A, Weimerskirch H. Variability in foraging behaviour of red-footed boobies nesting on Europa Island. Acta Oecologica. 2016;72: 87–97. doi:10.1016/j.actao.2015.10.017

17. Mendez L, Borsa P, Cruz S, De Grissac S, Hennicke J, Lallemand J, et al. Geographical variation in the foraging behaviour of the pantropical red-footed booby. Mar Ecol Prog Ser. 2017;568: 217–230. doi:10.3354/meps12052
